# The MYC/TXNIP axis mediates NCL-Suppressed CD8^+^T cell immune response in lung adenocarcinoma

**DOI:** 10.1186/s10020-025-01224-3

**Published:** 2025-05-09

**Authors:** Dan Xiao, Tanxiu Chen, Xinlin Yu, Ying Song, Yigang Liu, Wei Yan

**Affiliations:** 1 Department of Thoracic Oncology, Jiangxi Cancer Hospital＆Institute, Jiangxi Clinical Research Center for Cancer, The Second Affiliated Hospital of Nanchang Medical College, Jiangxi Key Laboratory of Oncology, No. 519 Beijing East Road, Nanchang, 330029 Jiangxi China; 2 Jiangxi Academy of Clinical Medical Sciences, The First Affiliated Hospital, Jiangxi Medical College, Nanchang University, Nanchang, 330006 China; 3 Department of Medical Laboratory, Jiangxi Cancer Hospital＆Institute, Jiangxi Clinical Research Center for Cancer, The Second Affiliated Hospital of Nanchang Medical College, Jiangxi Key Laboratory of Oncology, No. 519 Beijing East Road, 330029 Nanchang, China; 4 Department of Ultrasound Medicine, Jiangxi Cancer Hospital＆Institute, Jiangxi Clinical Research Center for Cancer, The Second Affiliated Hospital of Nanchang Medical College, Jiangxi Key Laboratory of Oncology, No. 519 Beijing East Road, Nanchang, 330029 Jiangxi China

**Keywords:** Lung adenocarcinoma, CD8^+^ T cells, Glucose metabolism, Immune escape, NCL, MYC

## Abstract

**Background:**

Lung adenocarcinoma is a deadly malignancy with immune evasion playing a key role in tumor progression. Glucose metabolism is crucial for T cell function, and the nucleolar protein NCL may influence T cell glucose metabolism. This study aims to investigate NCL’s role in T cell glucose metabolism and immune evasion by lung adenocarcinoma cells.

**Methods:**

Utilizing single-cell RNA sequencing (scRNA-seq) data from the Gene Expression Omnibus (GEO) and The Cancer Genome Atlas (TCGA), we analyzed cell clustering, annotation, and prognosis. In vitro experiments involved manipulating NCL expression in CD8^+^ T cells to study immune function and glucose metabolism. In vivo studies using an orthotopic transplant mouse model monitored NCL’s impact on CD8^+^ T cell glucose metabolism and anti-tumor immune function.

**Results:**

NCL was associated with T cell dysfunction and glucose metabolism. NCL silencing enhanced CD8^+^ T cell glucose metabolism, cytotoxicity, and infiltration, while NCL overexpression had the opposite effect. NCL overexpression relieved MYC-mediated transcriptional repression of TXNIP, reducing CD8^+^ T cell glucose metabolism. In vivo, NCL inhibited CD8^+^ T cell glucose metabolism through the MYC/TXNIP axis, hindering anti-tumor immune function.

**Conclusions:**

NCL overexpression suppresses CD8^+^ T cell glucose metabolism and anti-tumor immune function, promoting lung adenocarcinoma progression via the MYC/TXNIP axis.

**Supplementary Information:**

The online version contains supplementary material available at 10.1186/s10020-025-01224-3.

## Introduction

Lung adenocarcinoma, a common malignant tumor with a high mortality rate worldwide, has attracted significant attention (Wang et al. 2022a, b, c, 2023a, b; Zhang et al. 2023a, b). During the development of lung adenocarcinoma, tumor immune escape plays a crucial role, with the abnormal function of T cells considered one of the key mechanisms of immune escape in lung adenocarcinoma (Park et al. 2021; Wang et al. 2021). Particularly, CD8^+^ T cells, as important antigen-specific cells with cytotoxic effects, play an indispensable role in the immunotherapy of lung adenocarcinoma (Guo et al. 2018; Duhen et al. 2018). By recognizing and killing cancer cells, CD8^+^ T cells play a significant role in the anti-tumor immune response in patients with lung adenocarcinoma (Liu et al. 2023a, b; Song et al. 2022a, b; Sun et al. 2022). Nevertheless, various immune inhibitory factors exist within the lung adenocarcinoma environment, such as immune suppressive factors in the tumor microenvironment and overexpression of PD-L1, which may lead to the inhibition and exhaustion of CD8^+^ T cell function, thereby weakening their anti-tumor effects (Liu et al. 2022; Zhang et al. 2021; Li et al. 2022a, b). Therefore, therapeutic strategies targeting lung adenocarcinoma immune escape need to comprehensively consider how to activate and enhance the activity of CD8^+^ T cells to improve the effectiveness of immunotherapy, bringing about better treatment outcomes and quality of life for patients with lung adenocarcinoma (Wang et al. 2022a, b, c; Duan et al. 2021; Zhang et al. 2023a, b).

Glucose metabolism plays a critical role in regulating the function of immune cells, particularly for the optimal functioning of T cells (Wicks and Semenza 2022; Benesova et al. 2022). Glucose, as the primary energy source required for T cell growth, proliferation, and immune responses, is essential for maintaining normal T cell functions (Santos et al. 2021). Abnormalities in glucose metabolism can interfere with the biological activity of T cells, leading to functional impairments and inadequate immune responses against tumor cells, thus promoting tumor development and immune escape. In-depth research on the role of glucose metabolism in tumor immune regulation is crucial for elucidating the immune escape and tolerance mechanisms of lung adenocarcinoma (Gähler et al. 2022). By investigating the role of glucose in the tumor microenvironment, we can gain a better understanding of the bottlenecks in immunotherapy for lung adenocarcinoma, laying the theoretical foundation for developing targeted therapies against glucose metabolism to enhance the effectiveness of immunotherapy and improve survival rates for patients with lung adenocarcinoma (Tang et al. 2019).

Nucleolin (NCL), a multifunctional protein in cell biology, participates in multiple important biological processes both inside and outside the cell nucleus (Sen Gupta et al. 2018). Recent studies suggest that NCL may play a significant role in regulating glucose metabolism, providing cells with a new energy source and regulatory signals (Schwab et al. 2023; Wang et al. 2022a, b, c). However, the exact impact of NCL on the immune escape mechanisms of CD8^+^ T cells in lung adenocarcinoma remains unclear, making it a topic of considerable research interest (Seephan et al. 2023; Chen et al. 2023). The aim of this study is to delve into the specific mechanisms by which NCL operates in immune escape in lung adenocarcinoma and explore how it may affect the immune functions of CD8^+^ T cells through the regulation of glucose metabolism. By revealing the role of NCL in immune regulation of lung adenocarcinoma, we hope to provide new theoretical insights for the development of immunotherapy strategies for lung adenocarcinoma and offer new perspectives for the development of targeted therapeutic drugs against NCL, ultimately making a positive impact on the treatment of lung adenocarcinoma patients (Sui et al. 2021; Zhang et al. 2023a, b).

This study integrates Single-cell RNA Sequencing (scRNA-seq) data, bioinformatics analysis, in vitro experiments, and in vivo animal models to systematically investigate the regulatory impact of NCL on glucose metabolism and immune function of CD8^+^ T cells in lung adenocarcinoma. Specifically, the research will delve into how NCL modulates tumor immune escape through the MYC/TXNIP axis, elucidating the molecular mechanisms of NCL in the immune evasion process in lung adenocarcinoma (Song et al. 2022a, b; Seephan et al. 2023). By combining these research methodologies, we aim to reveal new functions and potential clinical significance of NCL in lung adenocarcinoma, providing crucial insights to deepen the understanding of tumor immune therapy mechanisms (Seephan et al. 2023; Chen et al. 2023). The outcomes of this study are expected to offer scientific foundation and clinical guidance for optimizing personalized treatment plans and immunotherapeutic strategies for lung adenocarcinoma patients, paving the way for improved therapeutic outcomes and patient survival rates, thus bringing renewed hope and progress to individuals with this condition (Seephan et al. 2023; Chen et al. 2023).

This study aims to explore in-depth the regulatory mechanisms of NCL on CD8^+^ T cell immune escape in lung adenocarcinoma, shedding light on its crucial role at the molecular level during the tumor immune evasion process. By conducting a detailed examination of NCL’s functions and mechanisms, we aim to provide an essential scientific basis for further understanding the immune escape mechanisms in lung adenocarcinoma, potentially discovering novel therapeutic targets and strategies. By uncovering the role of NCL in lung adenocarcinoma, this research will offer fresh perspectives and support for optimizing tumor immune therapy and devising personalized treatment plans, thereby opening up new avenues to enhance patient survival rates and treatment efficacy. Ultimately, we anticipate that the findings of this study will bring more hope and tangible benefits to lung adenocarcinoma patients, making valuable contributions to the advancement of lung adenocarcinoma treatment and the development of immunotherapy.

## Materials and methods

### Public data download

The scRNA-seq dataset GSE164789 related to Lung adenocarcinoma was obtained from the Gene Expression Omnibus (GEO). Simultaneously, the RNA-seq data was downloaded and curated from the STAR pipeline of the Cancer Genome Atlas (TCGA)-Lung adenocarcinoma project from TCGA database (https://portal.gdc.cancer.gov), extracting the data in Transcripts Per Million (TPM) format along with clinical information (Gu et al. 2022). As these data were derived from publicly available databases, no ethical committee approval was required (Table S1).

### scRNA-seq data analysis

The scRNA-seq data (GSE164789) was analyzed using the R software package Seurat (Butler et al. 2018). Initially, we filtered cells based on the following criteria: the number of expressed genes per cell (nFeature_RNA) was between 200 and 5000, the number of detected RNAs per cell (nCount_RNA) ranged from 1000 to 20,000, and the percentage of mitochondrial genes (percent.mt) in a single cell was less than 20%. Batch effects were then removed using canonical correlation analysis (CCA). Following normalization with the LogNormalize function, the top 2000 variable genes were subjected to Principal Component Analysis (PCA) using the RunPCA function, and the significant principal components were selected by JackStrawPlot and ElbowPlot functions for Uniform Manifold Approximation and Projection (UMAP) clustering analysis. Marker genes for each cell cluster were identified using the FindAllMarkers function, and cell types were manually annotated based on known cell marker genes (Shao et al. 2020).

Differential analysis of T cells between the Normal group and AIS group, AIS group and log_2_FCMIA group, and MIA group and IAC group was performed using the limma R package (Ritchie et al. 2015). Differential expression genes (DEGs) were selected using a threshold of |avg_| > 0.25 and *P*-value < 0.05, and the intersection of DEGs among the three groups was identified to obtain the overlapping genes.

### Transcriptome High-Throughput sequencing and data analysis

CD8^+^ T cell samples were collected from the sh-NC group (*n* = 3) and the sh-NCL-3 group (*n* = 3) for transcriptome high-throughput sequencing. The specific procedures were as follows: total RNA from each sample was extracted using Trizol reagent (T9424, Sigma-Aldrich, USA) following the manufacturer’s instructions. The RNA concentration, purity, and integrity were assessed using the Qubit^®^ RNA Analysis Kit (Q32852, Life Technologies, USA) in the Qubit^®^ 2.0 Fluorometer^®^, Nanodrop spectrophotometer (IMPLEN, Germany), and RNA Nano 6000 Analysis Kit (5067 − 1512, Agilent Technologies, USA) in the Bioanalyzer 2100 system to ensure an A260/280 ratio between 1.8 and 2.0. Each sample had a total RNA content of 3 µg as the input material for RNA sample preparation. Following the manufacturer’s recommendations, cDNA libraries were prepared using the NEBNext^®^ UltraTM RNA Library Prep Kit for Illumina (E7760S, New England Biolabs, China) and the library quality was assessed on the Agilent Bioanalyzer 2100 system. Subsequently, index-coded samples were clustered using TruSeq PE Cluster Kit v3 cBot HS (Illumina) on the cBot cluster generation system as per the manufacturer’s instructions. Post clustering, library preparation was sequenced on the Illumina HiSeq 550 platform, producing 125 bp/150 bp paired-end reads (Hong et al. 2020).

### Sequence data quality control and differential analysis

The quality of paired-end reads in the raw sequencing data was assessed using FastQC (v0.11.8). Cutadapt (v1.18) was then employed to preprocess the raw data by removing Illumina sequencing adapters and poly(A) tail sequences. A Perl script was utilized to eliminate reads with an N content exceeding 5%. Subsequently, the FASTX Toolkit (v0.0.13) was applied to extract reads with a base quality of 20 or higher, constituting 70% of the bases. Repair of the paired-end sequences was conducted using BBMap (v39.01). Ultimately, the filtered high-quality read segments were aligned to the human reference genome using HISAT2 (v0.7.12). Leveraging high-throughput transcriptome sequencing data, the R limma package was employed to identify differentially expressed genes between the sh-NC and sh-NCL-3 groups, with selection criteria set at |log_2_FC| > 2 and *P* < 0.05 (Wang et al. 2022a, b, c).

### Gene functional enrichment analysis

To further elucidate the specific functions of intersecting or differentially expressed genes, the clusterProfiler R software package (Yu et al. 2012) was used for analysis. Significance was set at *P* < 0.05 for conducting Gene Ontology (GO) and Kyoto Encyclopedia of Genes and Genomes (KEGG) enrichment analyses on the intersecting genes. GO categories encompassed Biological Processes (BP), Cellular Components (CC), and Molecular Functions (MF). Distinct visualizations like bar graphs, network diagrams, and cluster dendrograms were created for comprehensive analysis.

### Prognosis analysis

Utilizing the TCGA-lung adenocarcinoma dataset, a univariate COX analysis was conducted on the intersection genes. The R package “survival” was employed for proportional hazards assumption testing and survival regression fitting. Visualization of the results was carried out using the R packages “survminer” and “ggplot2”. The prognostic parameters examined encompassed Overall Survival, Disease-Specific Survival, and Progress Free Interval, with the statistical method applied to be the Logrank test. Survival curves for individual genes were plotted to compare the survival or disease progression disparities between high and low-expression groups, with a significance threshold set at *P* < 0.05 (Zhao et al. 2021).

Moreover, utilizing the TCGA-lung adenocarcinoma dataset, an analysis was conducted on the expression levels of key genes in normal lung tissues and tumor tissues of lung adenocarcinoma patients. The R package “pROC” was utilized for ROC analysis to plot ROC curves predicting the survival status of lung adenocarcinoma patients based on these key genes. Furthermore, the area under the curve (AUC) was calculated to predict the 1-10-year survival rates of lung adenocarcinoma patients (Cao et al. 2022).

### Establishment of animal models

Male BALB/c nude mice aged 6 weeks old, weighing 16–20 g, were purchased from Beijing Vital River Laboratory Animal Technology Co., Ltd. (Beijing, China). These mice were raised in specific pathogen-free conditions, with humidity maintained at 45–50% and temperature at 25–27℃, under a 12-hour light-dark cycle for one week prior to experimentation. The mice were fasted for 12 h prior to drug administration, with ad libitum access to food and water at other times. This study adhered to the principles of animal experimentation ethics, with all experiments receiving approval from the Ethics Committee of Jiangxi Cancer Hospital (No. 2023ky188), and conducted in accordance with international ethical guidelines. Every effort was made to minimize the animals’ pain and discomfort to ensure their well-being and welfare.

A lung adenocarcinoma orthotopic transplantation model was established by injecting LA795 cells (2 × 10^6^) and CMT64 cells (2 × 10^6^) into the left lung lobe of the nude mice at 6 weeks of age (Sievers et al. 2005). These tumor cells were labeled with the near-infrared fluorescent probe DiR (MX4005, Mocanbiotech, Shanghai, China) with a labeling efficiency of 90%. Subsequently, the bioluminescent signals of LA795 and CMT64 cells were analyzed using the CRi Maestro in vivo imaging system (Cambridge Research & Instrumentation, Massachusetts, USA). Near-infrared imaging technology was employed to quantitatively locate CD8^+^ T cells. Similarly, CD8^+^ T cells were first labeled with the near-infrared fluorescent probe DiR with a labeling efficiency of 93%, then injected (1 × 10^7^) into the mice intratumorally or intravenously 24 days post-tumor transplantation, followed by anesthesia and scanning using the CRi Maestro in vivo imaging system (Zhao et al. 2015).

After 28 days post CD8^+^ T cell injection, mice were anesthetized, euthanized by cervical dislocation, and tumor volume was calculated using the formula V = (length × width^2^)/2. Subsequently, tumor tissues were isolated and processed for downstream experimental analysis using FFPE (Formalin-Fixed Paraffin-Embedded) or rapid freezing.

The animals were grouped as follows: sh-NC group (injected with wild-type CD8^+^ T cells), sh-NCL-2 group (injected with CD8^+^ T cells with silenced NCL-2), sh-NCL-3 group (injected with CD8^+^ T cells with silenced NCL-3), oe-NC + oe-NC + oe-NC group (injected with CD8^+^ T cells transfected with lentivirus empty vector), oe-NCL + oe-NC + oe-NC group (injected with CD8^+^ T cells transfected with lentivirus overexpressing NCL and empty vector), oe-NCL + oe-MYC + oe-NC group (injected with CD8^+^ T cells transfected with lentivirus overexpressing NCL and MYC as well as empty vector), and oe-NCL + oe-MYC + oe-TXNIP group (injected with CD8^+^ T cells transfected with lentivirus overexpressing NCL, MYC, and TXNIP).

### 18 F-FDG PET/CT

The mice in each group were fasted for 8 h prior to the experiment. They were then intravenously injected with 290–320 µCi of 18 F-FDG (provided by the PET-CT center with a radiochemical purity > 97%). Thirty minutes later, pentobarbital sodium at a dose of 35 mg/kg was injected into the abdominal cavity of the mice. The mice were gently pressed to empty their bladders, then placed in a prone position and secured on a scanning bed with a warming device for PET/CT imaging. The small animal PET/CT imaging system, Inveon microPET/CT, was acquired from Siemens. The maximum uptake rate per gram of tissue in the lungs was measured using a region-of-interest (ROI) technique, calculated as the percentage of injected dose per gram (%ID/g) = ROI radioactivity/total injected radioactivity × 100% (Sijbesma et al. 2021).

### Immunohistochemical staining

Tumor tissues from various groups of mice were embedded in paraffin and sectioned. The sections were baked at 60℃ for 20 min and then sequentially deparaffinized in xylene, with two changes of xylene and 15 min of each immersion. Subsequently, they were immersed in absolute alcohol for 5 min, followed by another 5-minute change of absolute alcohol. The sections were then rehydrated in 95% and 70% alcohol for 10 min each. A drop of 3% H_2_O_2_ was added to each slide and left to incubate at room temperature for 10 min to block the endogenous peroxidase activity. Citrate buffer was added, and the slides were microwaved for 3 min, followed by incubation with antigen retrieval solution for 10 min at room temperature, then washed thrice with PBS. Normal goat serum blocking solution (E510009, Shanghai GenePharma Co., Ltd) was added and incubated at room temperature for 20 min. Subsequently, the slides were separately incubated overnight at 4℃ with diluted primary antibodies Ki67 (ab16667, 1:200, Abcam, UK), NCL (ab129200, 1:500, Abcam, UK), GLUT1 (ab115730, 1:250, Abcam, UK), GLUT4 (ab313775, 1:5000, Abcam, UK), HK2 (ab209847, 1:500, Abcam, UK), LDHA (SAB2108169, 1:200, Sigma-Aldrich, USA), and LDHB (SAB2108609, 1:200, Sigma-Aldrich, USA). After washing with PBS three times, the slides were incubated with secondary goat anti-rabbit IgG antibody (ab6721, 1:1000, Abcam, UK) for 30 min, washed with PBS, and then developed with DAB staining kit (DAB-M, Sigma-Aldrich, USA) by adding drops of solutions A, B, and C onto the specimen for 6 min. Subsequently, the slides were counterstained with hematoxylin for 30 s, dehydrated in 70%, 80%, 90%, 95% ethanol, and absolute ethanol for 2 min each, followed by two 5-minute washes in xylene and mounted with neutral resin. The slides were observed using a brightfield microscope (BX63, Olympus, Japan). The experiment was repeated three times. A negative control was achieved by incubating with PBS instead of the primary antibody. Five immunohistochemical images from different fields were selected for quantitative analysis. The number of cells with brown staining signals in the cytoplasm in each field was counted as positive stained cells, and the total cell count in each field was determined. The percentage of positive cells was calculated as the ratio of brown-stained cells to total cells × 100% (Wang and Wu 2020).

### Flow cytometry

Using flow cytometry, NCL^+^CD4^+^ T cells, NCL^+^CD8^+^ T cells, and NCL^+^Treg cells were isolated from the tumor tissues of mice in each group. The procedure included staining CD3^+^ T cells with FITC-conjugated anti-CD4 (11-0041-82, Thermo Fisher, USA), APC-conjugated anti-CD8 (47-0081-82, Thermo Fisher, USA), PerCP-Cy5.5-conjugated anti-CD25 (45-0251-82, Thermo Fisher, USA), and PE-conjugated anti-NCL antibody (ab305946, Abcam, UK). NCL^+^CD8^+^, NCL^+^CD4^+^ cells, or NCL^+^Treg cells were sorted. Cells were then incubated for 30 min at 4 °C in the dark followed by the addition of 2 mL PBS (P4417, Sigma-Aldrich, USA) solution. After centrifugation at 1500×g for 10 min at 4 °C, the supernatant was discarded. The cells were fixed and permeabilized using the FoxP3 staining kit (00-5523-00, Thermo Fisher, USA) solution, incubated in the dark on ice with PE-Cy7-conjugated anti-FoxP3 (25-5773-82, Thermo Fisher, USA) for 30 min, and kept at 4 °C in the dark. Flow cytometry analysis was performed using the FACS Aria II flow cytometer (BD Biosciences, USA) within 24 h (He et al. 2017).

Flow cytometry was utilized to analyze the number of IFN-γ and GZMB-positive cells in CD8^+^T cells of different groups. The procedure entailed fixing and permeabilizing CD8^+^T cells with Cytofix/Cytoperm Plus kit (555028, BD, USA), followed by staining the cells with APC-conjugated anti-CD8 (47-0081-82, Thermo Fisher, USA), PE-GZMB (17-8898-82, Thermo Fisher, USA), and BV421-IFN-γ (563376, BD, USA) antibodies. Data from stained cells were collected and analyzed using a flow cytometer and Flowjo CE software (Luo et al. 2021).

Flow cytometry was employed to determine the percentage of IFN-γ-positive cells in CD8^+^ T cells from each mouse tumor group. Initially, CD3^+^T cells were stained with APC-conjugated anti-CD8 (47-0081-82, Sigma-Aldrich, USA) and PE-conjugated anti-IFN-γ antibodies to isolate IFN-γ^+^CD8^+^T cells. After a 30-minute incubation at 4℃ in darkness, 2 mL of PBS (P4417, Sigma-Aldrich, USA) solution was added. The cells were then fixed with a 2% paraformaldehyde (30525-89-4, Sigma-Aldrich, USA)/PBS solution, kept at 4℃ in darkness, and analyzed within 24 h using the FACS Aria II flow cytometer (BD Bioscience, USA) (He et al. 2017).

Flow cytometry was used to examine the JC-1 signal in CD8^+^T cells from different groups. The procedure involved culturing CD8^+^T cells in high-glucose RPMI-1640 medium (R8758, Sigma-Aldrich, USA, containing 4.5 g/L glucose) and adding JC-1 (ab113850, Abcam, UK) to the mitochondrial membrane potential assay kit. After 24 h of stimulation with CD3/CD28, cells were analyzed using flow cytometry, and the ratio of JC-1 red to JC-1 green was used to indicate the cells’ Δψm (Zhong et al. 2022).

Flow cytometry was utilized to analyze the apoptosis status of tumor cells from different groups. Tumor cells (1 × 10^5^/well) were collected, washed in chilled PBS, and stained in the dark for 15 min using the APOAF-20TST assay kit (Sigma-Aldrich, USA). The precipitate was resuspended in 400 µL of binding buffer, and 5 µL of Annexin-V staining provided by the kit was added. Cells were analyzed using a flow cytometer. Cells in the upper right quadrant with the phenotype Annexin V + PI + represented late apoptosis cells; those in the lower right quadrant with the phenotype Annexin V + PI- represented early apoptosis cells; cells in the upper left quadrant with the phenotype Annexin V-PI + were necrotic cells; and cells in the lower left quadrant with the phenotype Annexin V-PI- represented live cells (Zhou et al. 2021).

### Cell culture

CD8^+^ T cells were isolated from the spleens and lymph nodes of three 9-10-week-old male C57BL/6J mice (219, Beijing Vitonlihua, China) using a mouse CD8^+^ T cell isolation kit (70902-50, Beaverbio, Suzhou, China). The cells were stained with FITC-conjugated anti-mouse CD8 antibody (ab313759, Abcam, UK) before and after sorting and analyzed by flow cytometry, showing purity levels of 13.8% and 97.1%, respectively, indicating successful CD8^+^ T cell isolation. The supernatant was collected by centrifugation at 3000×g for 10 min and filtered through a 0.2 μm filter (Wang et al. 2019a, b). Isolated CD8^+^ T cells were then stimulated with 4 × 10^7^ beads/mL of CD3/CD28 mouse activator (11452D, Thermo Fisher, USA) in a ratio of 1:1 cell to bead for 24 h before the experiment.

The mouse lung adenocarcinoma cell lines LA795 (ml096545, Enzyme-linked Biotechnology, Shanghai, China) and CMT64 (ml096469, Enzyme-linked Biotechnology, Shanghai, China) were cultured in RPMI-1640 medium (R4130, Sigma-Aldrich, USA) containing 10% fetal bovine serum (FBS) (F8687, Sigma-Aldrich, USA). The cells were maintained at 37℃ in a humidified 5% CO_2_ atmosphere in a Heracell™ Vios 160i CR CO_2_ incubator (51033770, Thermo Scientific, USA) and passaged when reaching 80%~90% confluency. Co-culture experiments involved adding CD8^+^ T cells to E0771 culture flasks at a cell ratio of 5:1 and incubating the mixed cells in a CO_2_ incubator (Cheng et al. 2022).

HEK293T cells were obtained from ATCC (CRL-3216) and cultured in DMEM medium (11965092, Gibco, USA) supplemented with 10% FBS, 10 µg/mL streptomycin, and 100 U/mL penicillin. These cells were also maintained at 37℃ in a humidified 5% CO_2_ atmosphere and passaged at 80%~90% confluency (Lu et al. 2016).

### Silence and overexpression in lentivirus vector construction

Potential short hairpin RNA (shRNA) target sequences for NCL were designed based on the mouse cDNA sequence analysis from GenBank. Three sequences targeting NCL were initially designed, with one sequence lacking the interference sequence serving as a negative control (sh-NC), as shown in Table S2. These oligonucleotides were custom-synthesized by Sigma-Aldrich and used to build lentivirus interference vector LV-1 (pGLVU6/GFP) (C06001, Sigma-Aldrich, China) within the lentivirus packaging system.

The packaged virus and the target vector were co-transfected into human embryonic kidney cell line HEK293T using Lipofectamine 2000 (11668030, Thermo Fisher, USA) at a cell confluency of 80–90%. Cell culture supernatant was collected after 48 h, and the virus particles were isolated by filtration and centrifugation. Viral titers were determined by collecting the virus during the logarithmic growth phase. Lentiviruses overexpressing NCL, MYC, and TXNIP were constructed and packaged by Sigma-Aldrich, with the lentivirus overexpression vector being LV-PDGFRA (Zhang et al. 2019).

CD8^+^ T cells were grouped as follows: (1) sh-NC group (transfected with lentivirus silencing negative control), sh-NCL-2 and sh-NCL-3 group (transfected with lentivirus silencing NCL-2), oe-NC group (transfected with lentivirus overexpressing negative control), oe-NCL group (transfected with lentivirus overexpressing NCL); (2) oe-NC + oe-NC + oe-NC group (transfected with lentivirus overexpressing negative control), oe-NCL + oe-NC + oe-NC group (transfected with lentivirus overexpressing NCL and overexpressing negative control), oe-NCL + oe-MYC + oe-NC group (transfected with lentivirus overexpressing NCL, MYC, and overexpressing negative control), oe-NCL + oe-MYC + oe-TXNIP group (transfected with lentivirus overexpressing NCL, MYC, and TXNIP). Subsequently, the above-grouped CD8^+^ T cells were co-cultured with Lung adenocarcinoma cells for further analysis.

### Generation of multicellular spheroids (MCS) and their 3D Co-culture with CD8^+^ T cells

A total of 1000 cancer cells/spheroids (Sigma-Aldrich, USA, A6013) were seeded in 35 or 81-well agarose tubes, utilizing the 3D Petri dish system produced by Microtissues^®^, Inc. (RI, USA) for spheroid formation. One minute after cell seeding, 1 mL (for 35-well tubes) or 2 mL (for 81-well tubes) of cell culture medium was added to promote the formation of cancer spheroids at 37℃ under 5% CO_2_ conditions. The following day, CD8^+^ T cells labeled with carboxyfluorescein succinimidyl ester (CFSE, Sigma-Aldrich, USA, 150347-59-4) were added at a ratio of 5:1 to the lung adenocarcinoma cells (LA795 and CMT64) in the cancer spheroids, maintaining co-culture in RPMI medium containing 10% fetal bovine serum and 100 units/mL penicillin/streptomycin. After 24 h of co-incubation, the spheroids were washed at 37℃ and fixed with 4% paraformaldehyde, followed by imaging using a ZEISS ZEN 710 confocal microscope. Images were acquired at the center of the spheroids, and surface maps were generated using Image J software.

After 2 days of co-culture, invasion experiments with Type I collagen (CC050, Sigma-Aldrich, USA) were initiated. Initially, collagen was neutralized to pH 7.0–8.0 and embedded in the co-culture, followed by 1-hour incubation in inverted agarose tube molds. Subsequently, the tube molds were inverted again, and RPMI medium containing 5% FBS and 1% Penicillin/Streptomycin was added. Invasion assays were carried out over 2 days, with images captured using an inverted microscope (Caikon, XDS-900, Shanghai, China) until cell recovery from the collagen matrix was observed.

To assess the cytotoxicity of CD8^+^ T cells towards MCS, spheroids were washed after 24 h of co-culture, stained and fixed using a cell viability/cytotoxicity kit (Biotium, USA, 30002). Confocal microscopy was employed to capture images, performing Z-stack scans every 5 μm from the top to the middle of the MCS, followed by maximal intensity projection. A 2.5D surface display was generated using Zeiss imaging software. For quantification of live/dead cells, Image J software was utilized to measure the total cell area for each dye (Zhou et al. 2021; Lin et al. 2022).

### Chromatin Immunoprecipitation (ChIP) experiment

The ChIP assay was performed using the EZ-Magna ChIP TMA kit (17-10086, Sigma-Aldrich, USA) to examine the enrichment of NCL protein on the MYC promoter and the enrichment of MYC protein on the TXNIP promoter. Cells in the logarithmic growth phase were cross-linked with 1% formaldehyde for 10 min, followed by quenching the cross-linking with 125 mM glycine at room temperature for 5 min. The cells were then washed twice with pre-chilled PBS, collected by centrifugation at 2000 g for 5 min, and resuspended in cell lysis buffer to a final concentration of 2 × 10^6^ cells per 200 µL. A protease inhibitor cocktail was added, followed by centrifugation at 5000 g for 5 min. The pellet was resuspended in nuclear lysis buffer, incubated on ice for 10 min, and sonicated to obtain chromatin fragments of 200–1000 bp. The lysate was centrifuged at 14,000 g at 4℃ for 10 min, and the supernatant was collected. Each sample of 100 µL of supernatant (containing DNA fragments) was mixed with 900 µL of ChIP Dilution Buffer and 20 µL of 50× Protein Inhibitor Cocktail (PIC). Subsequently, 60 µL of Protein A Agarose/Salmon Sperm DNA was added to each sample. After rotating at 4℃ for 1 h, the samples were centrifuged at 700 g for 1 min at 4℃. The supernatant was collected, with 20 µL reserved as input. For the experimental groups, the supernatant was incubated separately with 1 µL of NCL rabbit antibody (N2662, Sigma-Aldrich, USA) or MYC rabbit antibody (06-340, Sigma-Aldrich, USA) for the positive control or 1 µL of rabbit IgG antibody (ab172730, Abcam, UK) for the negative control, each supplemented with 60 µL of Protein A Agarose/Salmon Sperm DNA. After rotating at 4℃ for 2 h, the samples were centrifuged at 700 g for 1 min. The supernatant was discarded, and the pellet was washed successively with low salt buffer, high salt buffer, LiCl solution, and TE buffer (2 times). Each sample was washed twice with 250 µL of ChIP Wash Buffer. The DNA-protein cross-links were reversed using 20 µL of 5 M NaCl, and the DNA was extracted for further analysis using fluorescence quantitative PCR to detect the enriched chromatin fragments (Li et al. 2019). Specific primers for the MYC gene promoter region were as follows: Forward: AGTCTTTGATATGGAGACAGACTAG; Reverse: GAGCCACCATACCAAGCCTGTTTGC (Wu et al. 2020). Specific primers for the TXNIP gene promoter region were as follows: Forward: CAGAGCGCAACAACCATT; Reverse: AGGCTCGTGCTGCCCTCGTGCAC (Ji et al. 2016).

### Dual-Luciferase reporter assay experiment

Dual-luciferase reporter gene vectors were constructed for the target genes MYC and TXNIP, along with mutant variants with mutations at the binding sites for NCL (MYC-WT and MYC-MUT) and MYC (TXNIP-WT and TXNIP-MUT) respectively (Wu et al. 2020; Ji et al. 2016). The constructed plasmids were co-transfected into HEK-293T cells along with overexpression plasmids and negative control plasmids. After 48 h of transfection, the cells were harvested and lysed, and the supernatant was collected by centrifugation at 12,000 g for 1 min. The Dual-Luciferase Reporter Assay System (E1910) was employed to measure luciferase activity. Each cell sample was treated with 100 µL of Firefly luciferase working solution to detect Firefly luciferase activity and 100 µL of Renilla luciferase working solution to detect Renilla luciferase activity. The ratio of Firefly luciferase to Renilla luciferase was used as a measure of luciferase activity. Each experimental group was repeated three times for statistical analysis.

### Reverse transcription quantitative polymerase chain reaction (RT-qPCR)

Total RNA was extracted using the Trizol reagent kit (T9424, Sigma-Aldrich, USA). The quality and concentration of RNA were assessed by a UV-visible spectrophotometer (ND-1000, Nanodrop, Thermo Fisher, USA). Reverse transcription was performed according to the PrimeScript™ RT-qPCR kit (RR086A, TaKaRa, Mountain View, USA). Real-time quantitative reverse transcription polymerase chain reaction (RT-qPCR) was conducted on the LightCycler 480 system (Roche Diagnostics, Pleasanton, USA) using SYBR Premix Ex TaqTM (DRR820A, TaKaRa, Japan). GAPDH was utilized as the internal reference for mRNA. The primer design was outsourced to Shanghai General Biotech Co., Ltd., and the primer sequences are available in Table S3. The fold change in gene expression between the experimental and control groups was calculated using the 2-ΔΔCT method, where ΔΔCT = ΔCt experimental group - ΔCt control group, with ΔCt defined as the Ct of the target gene minus the Ct of the internal reference gene (Wang et al. 2019a, b).

### Western blot

The efficient RIPA lysis buffer (R0010) from Solarbio Corporation, located in Beijing, China, was used for the extraction of total cellular or tissue proteins following the manufacturer’s instructions. After 15 min of lysis at 4℃, the lysate was centrifuged at 12,000 × g for 15 min, and the supernatant was collected for protein concentration measurement using the BCA assay kit (20201ES76) from Yisheng Biotechnology Co., Ltd., based in Shanghai, China. Protein quantification was performed based on different concentrations, and protein separation was carried out using polyacrylamide gel electrophoresis, followed by wet transfer of proteins onto a PVDF membrane. The membrane was then blocked with 5% BSA at room temperature for 1 h. The primary antibody (refer to Table S4 for antibody information) was incubated overnight at 4℃, followed by washing the membrane with TBST thrice for 5 min each and incubating with diluted HRP-conjugated goat anti-rabbit IgG (ab205718, 1:20000, Abcam, UK) at room temperature for 1 h. The membrane was washed again with TBST thrice for 5 min each and then incubated with a developer solution for visualization. Protein quantification analysis was performed using ImageJ software (v1.48, National Institutes of Health, USA) by calculating the ratio of the grayscale values of each protein to the internal control GAPDH (Wu and Yi 2018). The experiment was repeated three times.

### Cell viability assay using MTT

CD8^+^ T cells were seeded at a density of 1 × 10^4^ cells per well in a 96-well plate with culture medium and incubated for 24 h. Cells from different treatment groups were mixed with 0.01 mL of MTT solution (CT02, Sigma Aldrich, USA) and incubated in a culture chamber for 4 hours. Subsequently, 0.1 mL of isopropanol containing 0.04 N HCl was added to each well, and thorough mixing was achieved using a multi-channel pipette. The HCl converted the phenol red in the culture medium into a yellow color that did not interfere with the measurement of MTT formazan. Cell viability was determined by measuring the absorbance at 570 nm (Shen et al. 2019).

### Cell proliferation assay using CCK-8

Tumor cell viability was assessed using a CCK-8 assay kit (C0038, Shanghai Biyuntian Biotechnology Co., Ltd., Shanghai, China). After transfection, cells were detached after 48 h, resuspended in L15 and MEM culture media, and then seeded in a 96-well plate at a concentration of 3 × 10^3^ cells per well. Cell viability was assessed by measuring absorbance at 24, 48, and 72 h post-seeding. Subsequently, 10 µL of CCK-8 solution was added to each well in the 96-well plate. After incubation at 37℃ for 2 h, the absorbance at 450 nm was measured using a microplate reader (A51119500C, ThermoFisher, USA) (Xu et al. 2020). Each experiment was repeated three times.

### Enzyme-linked immunosorbent assay (ELISA)

The ELISA was performed to determine the levels of IFN-γ in the cell supernatant and the IFN-γ concentrations in the serum of each group of mice. The specific procedure is as follows: Initially, the antigen was diluted in coating buffer to an appropriate concentration and then added to the enzyme-labeled reaction well at 37℃ for a 40-minute incubation period. Subsequently, 5% fetal bovine serum (F8318, Wuhan Merck Biotechnology Co., China) was added. The diluted samples were then added to the enzyme-labeled reaction wells along with the enzyme-labeled antibody, substrate solution, and finally, 50 µL of stop solution per well to terminate the reaction. The experimental results were read at 450 nm using an ELISA reader (Bio-Rad, USA) within 20 min, a standard curve was plotted, and the data were analyzed (Zhang et al. 2020a, b).

### Glucose uptake and lactate content

To assess the glucose uptake ability of CD8^+^ T cells, each group of CD8^+^ T cells was incubated with the glucose derivative 2-DG (provided in the kit) at 37℃ for 20 min. The glucose uptake ability of the cells in each group was then tested according to the kit instructions (ab136955, Abcam, UK). Additionally, CD8^+^ T cells from each group were collected and washed with PBS, and the lactate content in the cells of each group was determined following the kit instructions (ab65330, Abcam, UK) (Beg et al. 2021).

### Adenosine triphosphate (ATP) quantification

CD8^+^ T cells (1 × 10^5^/well) were cultured in a 6-well plate and subjected to different treatments for incubation. The ATP levels were determined by cell lysis using an ATP assay kit (BC0300, Solarbio, Beijing, China) to measure total cellular ATP content. Following the manufacturer’s instructions, ATP extraction was performed from CD8^+^ T cells and measurements were carried out using a UV spectrophotometer (DU720, Beckman, USA) (Guo et al. 2021).

### Metabolic measurements

The Seahorse XFe96 Extracellular Flux Analyzer (Agilent Technologies) was employed for metabolic analysis. Extracellular acidification rate (ECAR) and oxygen consumption rate (OCR) of CD8^+^ T cells in each well were calculated. Cells were subjected to XF Glycolysis Stress Test or XF Cell Mito Stress Test with the following concentrations of injected compounds: 10 mM glucose (50-99-7, Sigma-Aldrich, USA), 2 µM oligomycin (1404-19-9, Sigma-Aldrich, USA), 50 mM 2-deoxy-D-glucose (2-DG) (154-17-6, Sigma-Aldrich, USA), 1 µM carbonyl cyanide 4-(trifluoromethoxy)phenylhydrazone (FCCP) (370-86-5, Sigma-Aldrich, USA), and 0.5 µM rotenone (83-79-4, Sigma-Aldrich, USA). XF Glycolysis Stress Test or XF Cell Mito Stress Test kits were purchased from Agilent Technologies, USA (Wu et al. 2022).

### Statistical analysis

Our study utilized R version 4.2.1 for programming within the integrated development environment of RStudio version 2022.12.0-353. Data processing was conducted using GraphPad Prism 8.0 software. Descriptive statistics were presented as mean ± standard deviation (Mean ± SD). The comparison between two groups was performed using an unpaired t-test, while multiple groups were compared using one-way analysis of variance (ANOVA). The homogeneity of variance was assessed through the Levene test. In cases of homogeneous variance, Dunnett’s T3 and LSD-t tests were employed for pairwise comparisons. In situations of heterogeneous variance, Dunnett’s T3 test was used. A p-value < 0.05 was considered statistically significant for comparing data between the two groups (Zhang et al. 2020a, b).

## Results

Single-cell Transcriptomics Reveal the Critical Role of T Cells in Lung Adenocarcinoma.

**Fig. 1:**
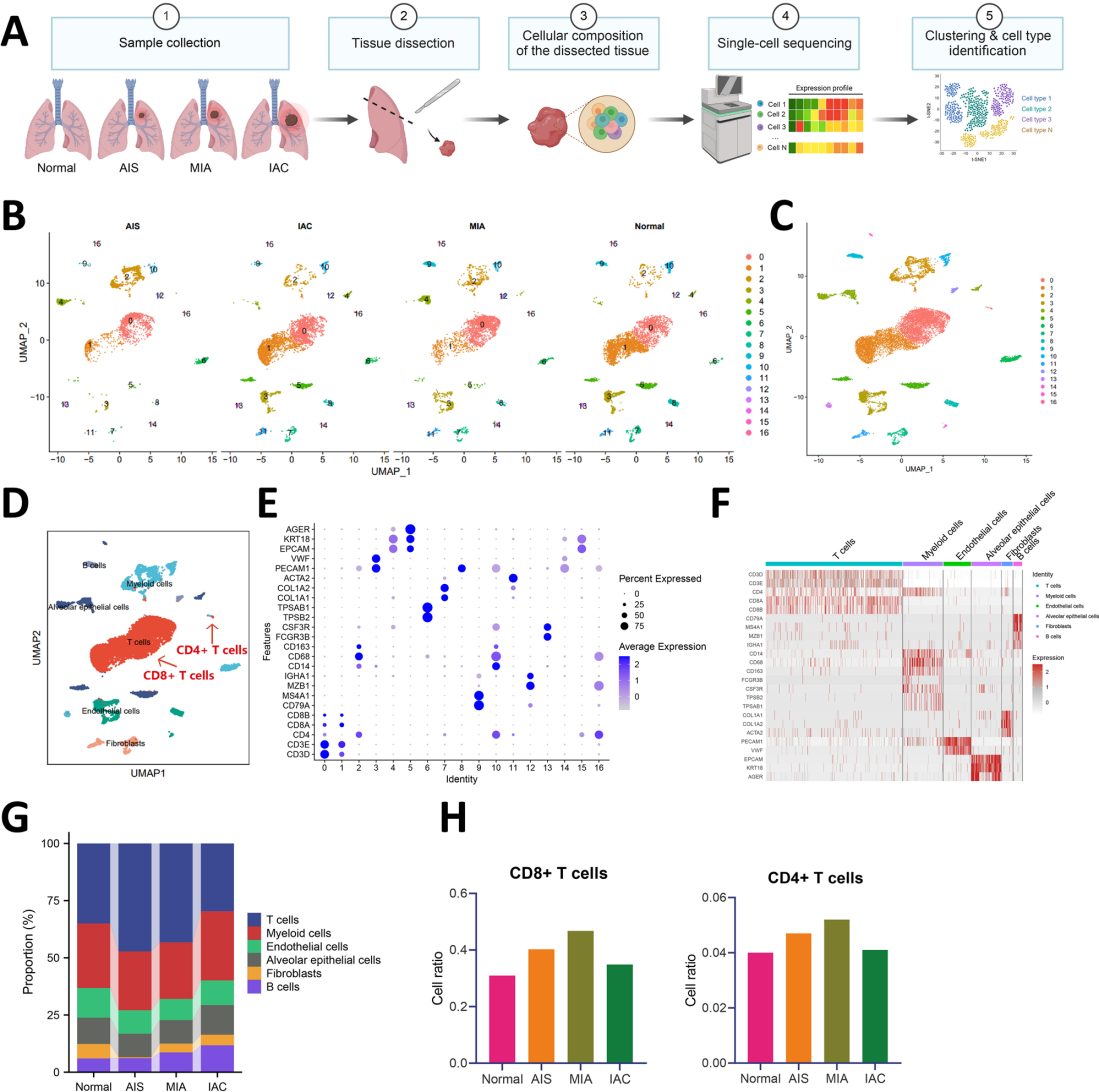
UMAP clustering analysis, cell annotation, and cell proportion analysis of scRNA-seq data. Notes: **(A)** Schematic of scRNA-seq data analysis workflow; **(B)** UMAP clustering analysis grouping cells into 17 cell clusters (displayed by sample source); **(C)** Overall representation of the 17 cell clusters; **(D)** Annotation of the 17 cell clusters into 6 cell types; **(E)** Bubble plot of marker genes for the 17 cell clusters; **(F)** Heatmap showing expression of marker genes in the 6 cell types; **(G)** Stacked bar plot comparing cell numbers across samples; **(H)** Variation in T cell numbers across samples

Lung adenocarcinoma accounts for about 40–55% of all lung cancer cases. Compared to lung squamous cell carcinoma, lung adenocarcinoma is often diagnosed at a later stage, with higher histological grades and an increased risk of recurrence, metastasis, and disease progression (Nguyen et al. 2022). In order to establish a systematic single-cell transcriptome map of lung adenocarcinoma, we downloaded the scRNA-seq dataset GSE164789 from the GEO database, comprising one sample of normal lung tissue (Normal group) and three samples of lung adenocarcinoma tissue from patients (including AIS for adenocarcinoma in situ, MIA for minimally invasive adenocarcinoma, and IAC for invasive adenocarcinoma) (Fig. 1A). AIS represents the earliest stage of adenocarcinoma, progressing to MIA with minimal invasion at the microscopic level and eventually to IAC, where the tumor exhibits full invasive capabilities (Zhu et al. 2022). This study aims to elucidate the cellular changes and molecular mechanisms involved in the occurrence and progression of lung adenocarcinoma based on single-cell mapping.

Initially, the data underwent quality control and standardization using the R package Seurat. Low-quality cells were filtered out, retaining cells expressing a gene count per single cell (nFeature_RNA) of > 200 & <5000, RNA counts per single cell (nCount_RNA) of > 1000 & <20,000, and with mitochondrial gene percentage (percent.mt) < 20, resulting in 15,258 single cells passing quality control (Figure S1A). The correlation analysis of post-filtering data showed a coefficient of -0.16 between nCount_RNA and percent. Mt and a coefficient of 0.91 between nCount_RNA and nFeature_RNA (Figure S1B), indicating good data quality post-filtering. Subsequently, highly variable genes were identified from the filtered cells, with the top 2000 variable genes selected for downstream analysis (Figure S1C).

Next, batch-corrected integration of all samples was performed, and PCA was conducted on the top 2000 variable genes using the RunPCA function. Cell cycle scores for the samples were computed using the CellCycleScoring function (Figure S1D), revealing no significant batch effects among the four samples, thus making them suitable for further analysis. A heatmap of the top 2 principal components was selectively visualized using the DimHeatmap function (Figure S1E), displaying the primary constituent genes of the first 2 principal components (Figure S1F). Visualizing the top 50 principal components using the JackStrawPlot function indicated that the P-values of the first 6 principal components were all < 0.05 (Figure S1G). Furthermore, combined with the ElbowPlot function, a change in standard deviation was observed at the 6th principal component (Figure S1H), suggesting that the first 6 principal components effectively captured the information contained in the selected highly variable genes.

Subsequently, non-linear dimensionality reduction using the UMAP algorithm was performed on the top 20 principal components, clustering all cells into 17 cell clusters (Fig. 1B-C). Based on marker genes, these clusters were annotated into 6 cell types: T cells, Myeloid cells, Endothelial cells, Alveolar epithelial cells, Fibroblasts, and B cells (Fig. 1D). We present UMAP expression maps of the six cell types. CD3D and CD3E represent markers for T cells, with CD4 being specific for CD4 + T cells (Cluster 0 and Cluster 1), and CD8A and CD8B serving as markers for CD8^+^ T cells (Cluster 16). This suggests that the predominant T cell subset in all samples is CD8^+^ T cells (Fig. 1E-F; Figure S2). Additionally, UMAP expression plots for these cell types were presented, showcasing specific marker genes such as CD3D, CD3E, CD4, CD8A, CD8B for T cells; CD79A, MS4A1, MZB1, IGHA1 for B cells; CD14, CD68, CD163, FCGR3B, CSF3R, TPSB2, TPSAB1 for Myeloid cells; COL1A1, COL1A2, ACTA2 for Fibroblasts; PECAM1, VWF for Endothelial cells; and EPCAM, KRT18, AGER for Alveolar epithelial cells (Fig. 1E-F; Figure S2). In this study, we provide a detailed description of the cellular composition distribution of six cell types across four sample types. Specifically, the quantity of T cells (primarily CD8^+^ T cells) shows significant variations in the occurrence and progression of lung adenocarcinoma. Compared to normal samples, early-stage lung adenocarcinoma samples exhibit a significant increase in the number of CD8^+^ T cells, while late-stage lung adenocarcinoma samples show a notable decrease in CD8^+^ T cell numbers (Fig. 1G-H).

These findings underscore the crucial role of T cells, especially CD8^+^ T cells, in the onset and progression of lung adenocarcinoma.

Transcriptional Study of T Cells Reveals Key Prognostic Gene NCL in Lung Adenocarcinoma Patients.

**Fig. 2:**
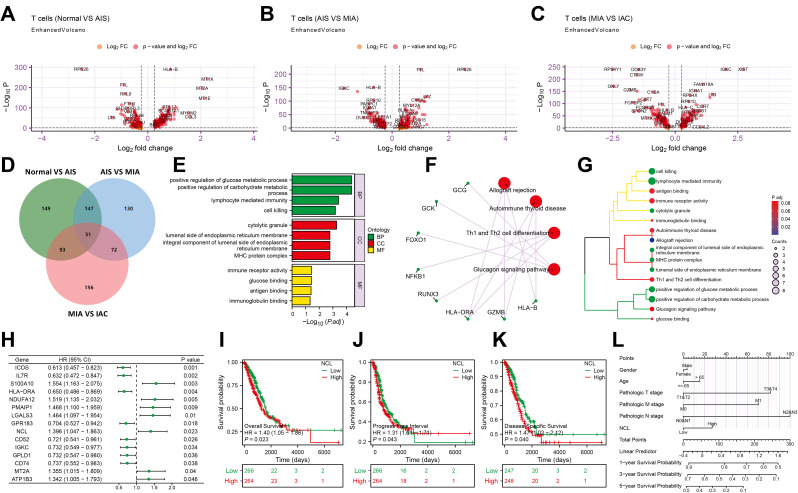
Identification of T cell marker genes associated with the prognosis of lung adenocarcinoma patients. Notes: **(A)** Volcano plot showing differential analysis between Normal group and AIS group; **(B)** Volcano plot showing differential analysis between AIS group and MIA group; **(C)** Volcano plot showing differential analysis between MIA group and IAC group; **(D)** Venn diagram illustrating the intersection of differential analysis results among the three groups; **(E)** Bar graph of GO terms associated with the intersecting genes, where BP denotes biological processes, CC signifies cellular components, and MF represents molecular functions; **(F)** Network diagram of KEGG pathways associated with the intersecting genes; **(G)** Cluster dendrogram of GO and KEGG enrichment analysis of the intersecting genes; **(H)** Forest plot predicting overall survival of lung adenocarcinoma patients based on 13 genes (*N* = 522); **(I-K)** Survival curve plots for high NCL expression and low NCL expression groups representing Overall Survival **(I)**, Disease-Specific Survival **(J)**, and Progress-Free Interval **(K)** of patients (*N* = 522); **(L)** Column chart depicting NCL expression predicting overall survival of lung adenocarcinoma patients (*N* = 522)

Using the threshold of |avg_log_2_FC| > 0.25 and *P* < 0.05, we identified a total of 440 differentially expressed genes (DEGs) between the Normal and AIS groups in T cells (Fig. 2A). Additionally, 400 DEGs were found between the AIS and MIA groups (Fig. 2B) and 372 DEGs between the MIA and IAC groups (Fig. 2C). Taking the intersection of these three sets of DEGs, we identified 51 common genes (Fig. 2D). GO and KEGG enrichment analyses revealed that these 51 genes are significantly enriched in pathways related to glucose metabolism and immune response (Fig. 2E-G). This suggests the crucial role of glucose metabolism in T cell immune function.

Furthermore, to pinpoint key genes, we conducted a single-factor COX analysis on the 51 common genes and identified 15 genes closely associated with the prognosis of Lung adenocarcinoma patients (Fig. 2H). Among these, only the expressions of GPLD1 and NCL were consistent with the prognosis trend. GPLD1 was found to be downregulated in the tumor group compared to the normal group, indicating it is a low-risk gene (Figure S3A; Fig. 2H). Conversely, NCL was upregulated in the tumor group and identified as a high-risk gene (Figure S3B; Fig. 2H). NCL demonstrated higher diagnostic and prognostic value in Lung adenocarcinoma patients compared to GPLD1 (Figure S3C-E). Analysis of the single-cell atlas showed a gradual decrease in GPLD1 expression in T cells from the Normal, AIS, MIA, to IAC groups (Fig. 2A-C), with relatively sparse expression and distribution across cells (Figure S3F). On the other hand, NCL exhibited a gradual increase in expression in T cells from the Normal, AIS, MIA, to IAC groups, with higher expression levels primarily in T cells (Figure S3G). Additionally, GPLD1 expression was found to be associated with Overall Survival in Lung adenocarcinoma patients (Figure S3H) while showing no significant difference in relation to Disease-Specific Survival and Progress-Free Interval (Figure S3I-J). NCL, however, was significantly associated with Overall Survival, Disease-Specific Survival, and Progress-Free Interval of Lung adenocarcinoma patients (Fig. 2I-K) and demonstrated a predictive value for patient prognosis to a certain extent (Fig. 2L). These results collectively indicate that the T cell marker gene NCL is a key gene influencing the prognosis of Lung adenocarcinoma patients.

Study on the Role of NCL in Immune Regulation of Tumor Cells by CD8^+^ T Cells.

**Fig. 3:**
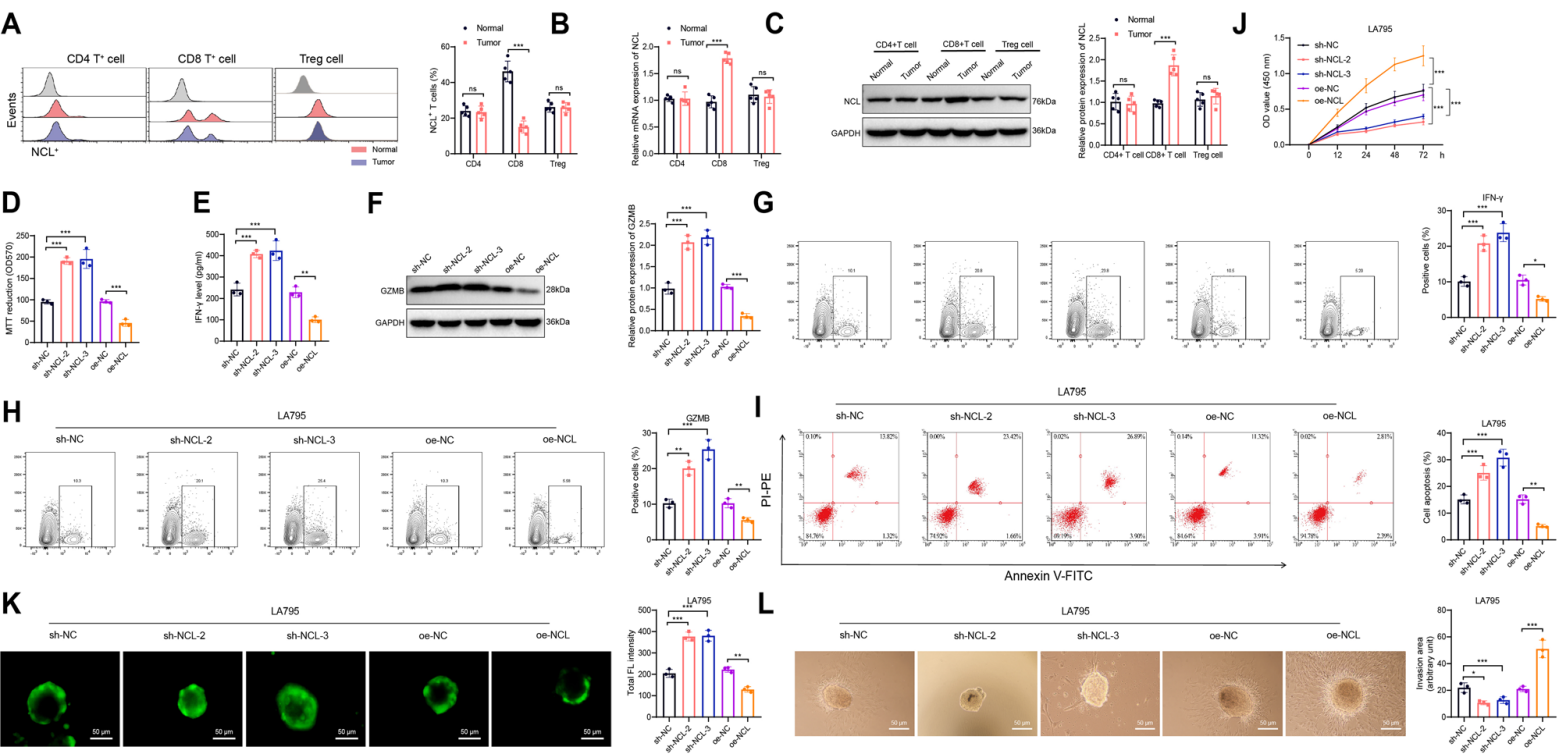
The investigation of NCL expression and function in CD8^+^ T cells and their impact on CD8 + T cell cytotoxicity against LA795 cells. Notes: **(A)** The isolation of NCL^+^CD4^+^ T cells, NCL^+^CD8^+^ T cells, and NCL + Treg cells from different tissues using flow cytometry and quantitative analysis of their numbers; **(B-C)** The detection of NCL expression levels in CD4^+^ T cells, CD8^+^ T cells, and Treg cells isolated from different tissues through RT-qPCR **(B)** and Western Blot **(C)**. **(D)** Cell activity was measured through MTT assay. **(E)** ELISA was employed to determine the levels of IFN-γ in the cell culture medium. **(F)** The abundance of GZMB in CD8 + T cells was detected via Western Blot. **(G-H)** Flow cytometry was utilized to analyze the quantities of IFN-γ-positive **(G)** and GZMB-positive **(H)** cells in CD8^+^ T cells and for subsequent quantitative analysis. **(I)** Flow cytometry was used to assess tumor cell apoptosis, with apoptotic cells marked by red boxes and their statistical data displayed in the accompanying graph. **(J)** CCK-8 assay was used to monitor the proliferation of tumor cells at different time points. **(K)** Confocal microscopy images demonstrated the infiltration of CFSE-labeled CD8^+^ T cells in MCS, with a scale bar of 50 μm, and the average CFSE fluorescence intensity **(L)** data for MCS. **(L)** Cell culture images were captured in bright field mode under inverted microscopy, outlining the invasive regions with a white dashed line, a scale bar of 50 μm, and statistical analysis of the invasive regions. All cell experiments were conducted thrice, with “ns” indicating *P* > 0.05, * denoting *P* < 0.05, ** denoting *P* < 0.01, and *** denoting *P* < 0.001

To delve deeper into the role of NCL in T cells, we established an in situ tumor model and utilized flow cytometry to analyze the quantities of NCL^+^CD4^+^ T cells and NCL^+^CD8^+^ T cells in both normal lung tissue and tumor tissue. The findings revealed a significant decrease in the number of NCL^+^CD8^+^ T cells in tumor tissue compared to normal lung tissue, while the quantities of NCL^+^CD4^+^ T cells and NCL^+^Treg cells showed no significant variation (Fig. 3A). Furthermore, we employed CD4 beads, CD8 beads, and Treg beads to purify immune cells isolated from the tissue. Subsequently, we examined the levels of NCL in these three cell types using RT-qPCR and Western Blot analysis. The results demonstrated a notable increase in NCL expression levels in isolated CD8^+^ T cells from tumor tissue as opposed to normal lung tissue, whereas the expression of NCL in CD4^+^ T cells and Treg cells showed no significant change (Fig. 3B-C). Therefore, CD8^+^ T cells were selected for further analysis.

In order to further investigate the role of NCL in CD8^+^ T cells, we isolated CD8^+^ T cells from the spleens and lymph nodes of normal mice. Subsequently, we either silenced or overexpressed NCL (Figure S4A). Three shRNA sequences targeting NCL were designed, and we confirmed the silencing efficiency of sh-NCL. Following validation, we selected sh-NCL-2 and sh-NCL-3, which demonstrated higher silencing efficacy, for subsequent experiments (Figure S4B-C). Additionally, we utilized lentiviral vectors to establish mouse-derived CD8^+^ T cells overexpressing NCL (oe-NCL, with oe-NC as the control). The transfection efficiency was confirmed using RT-qPCR and Western Blot techniques (Figure S4D-E).

CD8^+^ T cells from different genotypes were stimulated with CD3/CD28 for activation. Cell viability was assessed using the MTT assay 48 h post-stimulation, revealing a significant increase in viability in NCL-silenced cells and a decrease in viability in NCL-overexpressing cells (Fig. 3D). CD8^+^ T cells can secrete granzyme B (GZMB) and interferon-gamma (IFN-γ), enhancing their cytotoxicity against tumor cells (McKenzie et al. 2006). ELISA experiments demonstrated a significant increase in IFN-γ secretion in NCL-silenced cells and a decrease in IFN-γ levels in NCL-overexpressed cells (Fig. 3E). Western Blot analysis also showed a significant elevation of GZMB protein levels in NCL-silenced cells and a reduction in GZMB levels in NCL-overexpressed cells (Fig. 3F). Flow cytometry analysis confirmed a significant increase in IFN-γ and GZMB in NCL-silenced cells and a decrease in the overexpressed group (Fig. 3G-H). Overall, our study suggests that silencing NCL enhances the activity and cytotoxicity of CD8^+^ T cells, while NCL overexpression diminishes their functionality and cytotoxicity.

Subsequently, we further validated the impact of silencing or overexpressing NCL in CD8^+^ T cells on lung adenocarcinoma cells. CD8^+^ T cells from different groups were co-cultured with the lung adenocarcinoma cell lines LA795 or CMT64 after activation. Tumor cells were then isolated and their apoptosis was assessed using flow cytometry, while the proliferation capacity of the tumor cells was evaluated using the CCK-8 assay. The results revealed that compared to the sh-NC group, both the sh-NCL-2 and sh-NCL-3 groups exhibited increased tumor cell apoptosis and reduced proliferation capacity. In contrast, when compared to the oe-NC group, the oe-NCL group showed decreased tumor cell apoptosis and enhanced proliferation capacity (Fig. 3I-J, Figure S5A-B).

To mimic the in vivo environment, 3D cancer spheroids of LA795 and CMT64 cell lines were constructed, and CFSE-labeled CD8^+^ T cells were co-cultured. The permeability rate was significantly increased in the sh-NCL-2 and sh-NCL-3 group compared to the sh-NC group, while a decrease was observed in the oe-NCL group compared to the oe-NC group (Fig. 3K, Figure S5C), indicating that NCL influences the infiltration capacity of CD8^+^ T cells. Furthermore, after 4 days of co-culture, the invasive spread of spheroids was notably reduced in the presence of CD8^+^ T cells from the sh-NCL-2 and sh-NCL-3 group and enhanced in the oe-NCL group compared to their respective controls (Fig. 3L, Figure S5D). In conclusion, these experimental findings highlight the critical regulatory role of NCL in CD8^+^ T cells, significantly impacting the growth, invasion, and survival of tumor cells in their interaction.

NCL Inhibits CD8^+^ T Cell Glucose Metabolism and Affects Tumor Microenvironment Infiltration.

Previous studies have demonstrated the critical role of NCL in regulating the activity and cytotoxicity of CD8^+^ T cells. Literature has shown that glucose metabolism induces long-lasting immune activity, lifespan, and function in T cells (Patsoukis et al. 2015). Energy supply is a major factor controlling T-cell activity, particularly in tumor microenvironments with glucose restriction (Chang et al. 2015). Research has suggested that NCL may be involved in the glucose metabolism process (Schwab et al. 2023). Therefore, we investigated the impact of NCL on CD8^+^ T cell glucose metabolism.

**Fig. 4:**
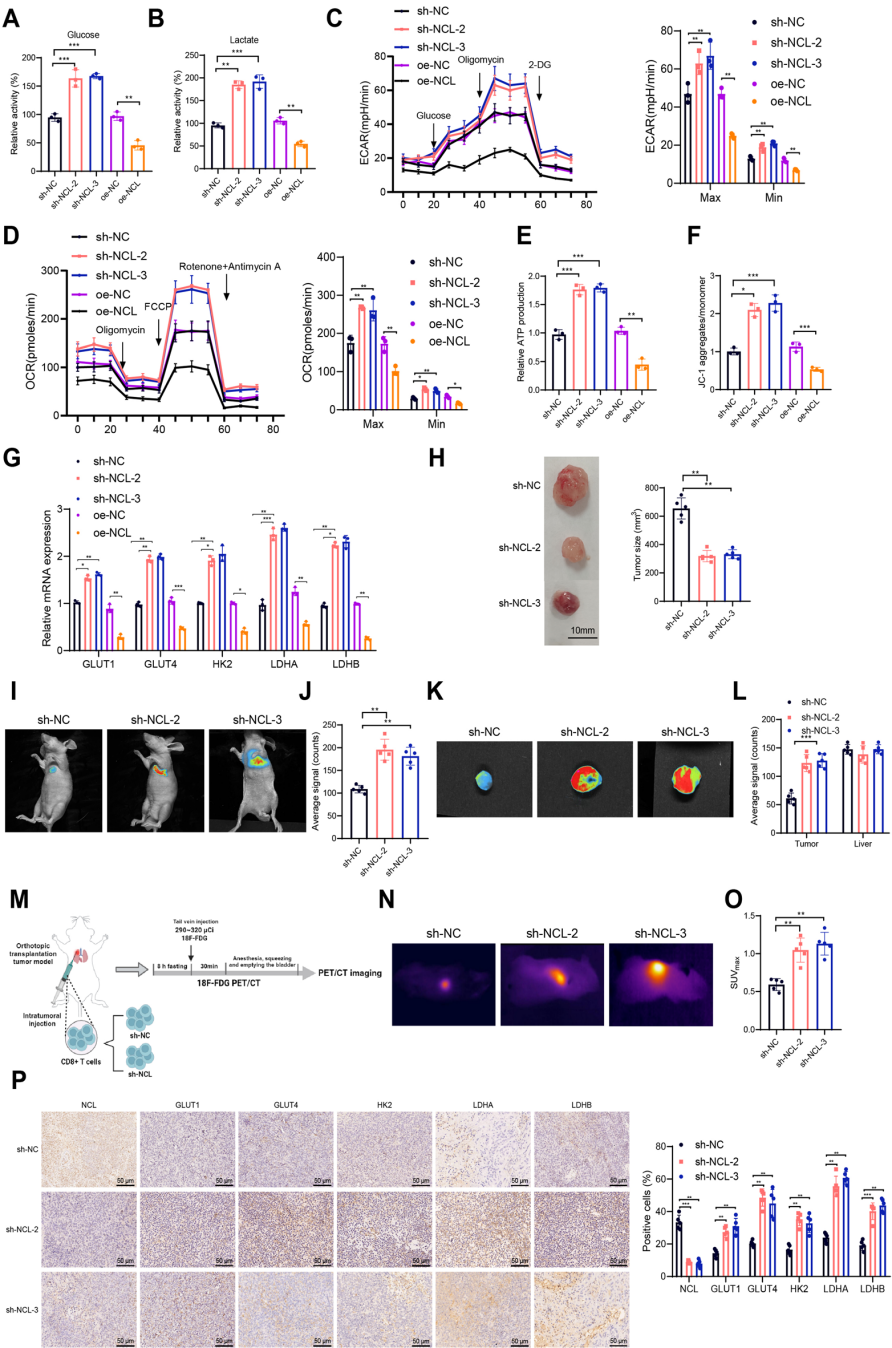
Impact of NCL on the reprogramming of glucose metabolism in CD8^+^ T cells. Notes: **(A)** Glucose uptake levels in CD8^+^ T cells of each group; **(B)** Lactate production levels in CD8 + T cells of each group; **(C)** Changes in ECAR of CD8^+^ T cells in each group, as well as minimum and maximum ECAR; **(D)** Changes in OCR of CD8^+^ T cells in each group, as well as minimum and maximum OCR; **(E)** Measurement of intracellular ATP in CD8^+^ T cells of each group using an ATP assay kit; **(F)** Detection of JC-1 signal in CD8^+^ T cells of each group by flow cytometry, calculation of the red-green signal ratio of JC-1 to determine mitochondrial membrane potential; **(G)** Expression levels of GLUT1, GLUT4, HK2, LDHA, and LDHB in CD8^+^ T cells of each group analyzed by RT-qPCR; **(H)** Morphological images of tumor tissues in each group of mice, along with volumetric statistics of the tumor tissues; **(I)** In vivo near-infrared imaging of mice injected with CD8^+^ T cells into the tumor six hours after injection; **(J)** Semi-quantitative visualization of CD8^+^ T cell signals in tumors; **(K-L)** In vitro images of tumors 24 h after injection of CD8^+^ T cells via tail vein **(K)**, semi-quantitative display of CD8^+^ T cell signals in tumors **(L)**; **(M)** Flow chart of animal experiments; **(N)** 18 F-FDG PET/CT images of mice in each group; **(O)** Quantitative statistics of SUVmax values in each group; **(P)** Immunohistochemical detection of positive expression of NCL, GLUT1, GLUT4, HK2, LDHA, and LDHB in tumor tissues of each group of mice, scale bar = 50 μm, including statistical charts of the ratio of positive cells; all cell experiments were repeated three times, * indicates *P* < 0.05, ** indicates *P* < 0.01, *** indicates *P* < 0.001

Initially, we observed that silencing NCL increased glucose uptake and lactate production in CD8^+^ T cells, while NCL overexpression had the opposite effect, indicating an inhibitory role of NCL in glycolysis (Fig. 4A-B). ECAR serves as another measurement of glucose metabolism, reflecting lactate-induced acidification of the surrounding medium in cancer cells (Schmidt et al. 2021). Results revealed that silencing NCL elevated ECAR in CD8^+^ T cells, with the converse observed in NCL overexpression, suggesting a potential inhibitory role of NCL in lactate formation during glycolysis (Fig. 4C). Furthermore, cellular oxygen consumption reflects mitochondrial respiration and can be measured through the OCR. The findings indicated that CD8^+^ T cells with silenced NCL exhibited higher OCR, while cells overexpressing NCL showed lower OCR, indicating that NCL acts as a negative regulator of basal mitochondrial respiration (Fig. 4D). Additionally, we analyzed the impact of NCL on ATP production and mitochondrial membrane potential, with consistent results showing that silencing NCL increased ATP production and mitochondrial membrane potential in CD8^+^ T cells, while NCL overexpression had the opposite effect (Fig. 4E-F). Further exploration of NCL’s role in glucose metabolism was conducted by detecting the expression of key enzymes in the glycolytic cascade using RT-qPCR. The results demonstrated significantly elevated expression of GLUT1, GLUT4, HK2, LDHA, and LDHB in the sh-NCL-2 and sh-NCL-3 group compared to the sh-NC group, whereas the oe-NCL group exhibited significantly reduced expression of these enzymes compared to the oe-NC group (Fig. 4G). These outcomes highlight the crucial role of NCL in CD8^+^ T cell glucose metabolism, where silencing NCL markedly enhances glycolysis and oxidative phosphorylation (OXPHOS), while NCL overexpression exerts the opposite effects.

Subsequently, we injected NCL-silenced CD8^+^ T cells into mice-bearing tumors to verify the impact of NCL silencing on in vivo CD8^+^ T cell activity and glucose metabolism. Our findings indicated that compared to the sh-NC group, the sh-NCL-2 and sh-NCL-3 groups (mice injected with NCL-silenced CD8^+^ T cells) exhibited smaller tumor volumes, suggesting the ability of NCL-silenced CD8^+^ T cells to inhibit tumor growth in vivo (Fig. 4H). Additionally, 24 h post-cell injection, we observed the accumulation of CD8^+^ T cells in tumors through near-infrared imaging. Results demonstrated a significant increase in CD8^+^ T cell accumulation in the sh-NCL-2 and sh-NCL-3 group compared to the sh-NC group (Fig. 4I-J). Furthermore, intravenous injection of CD8^+^ T cells from different groups followed by tumor excision after 24 h for ex vivo measurements revealed that the sh-NCL-2 and sh-NCL-3 groups had the highest accumulation of CD8^+^ T cells, indicating that silencing NCL significantly enhanced the infiltrative capacity of CD8^+^ T cells in the tumor microenvironment (Fig. 4K-L).

18 F-FDG PET/CT imaging reflects glucose metabolism in tumor tissues, with higher Maximum Standardized Uptake Value (SUVmax) values indicating increased metabolic activity. Our results showed that compared to the sh-NC group, the sh-NCL-2 and sh-NCL-3 groups exhibited significantly higher SUVmax values, predominantly distributed at the site of CD8^+^ T cell injection (Fig. 4M-O), suggesting that silencing NCL significantly promoted the uptake of 18 F-FDG by CD8^+^ T cells. Additionally, immunohistochemical analysis revealed decreased NCL expression and upregulated expressions of GLUT1, GLUT4, HK2, LDHA, and LDHB in the sh-NCL-2 and sh-NCL-3 groups compared to the sh-NC group (Fig. 4P). This indicates that silencing NCL significantly enhances the glucose metabolism activity of CD8^+^ T cells in vivo.

In conclusion, silencing NCL significantly enhances the glucose metabolism activity of CD8^+^ T cells, thereby augmenting their infiltration capacity in the tumor microenvironment.

Overexpression of NCL Releases MYC-Mediated Transcriptional Suppression of TXNIP to Inhibit Glucose Metabolism in CD8^+^ T Cells.

**Fig. 5:**
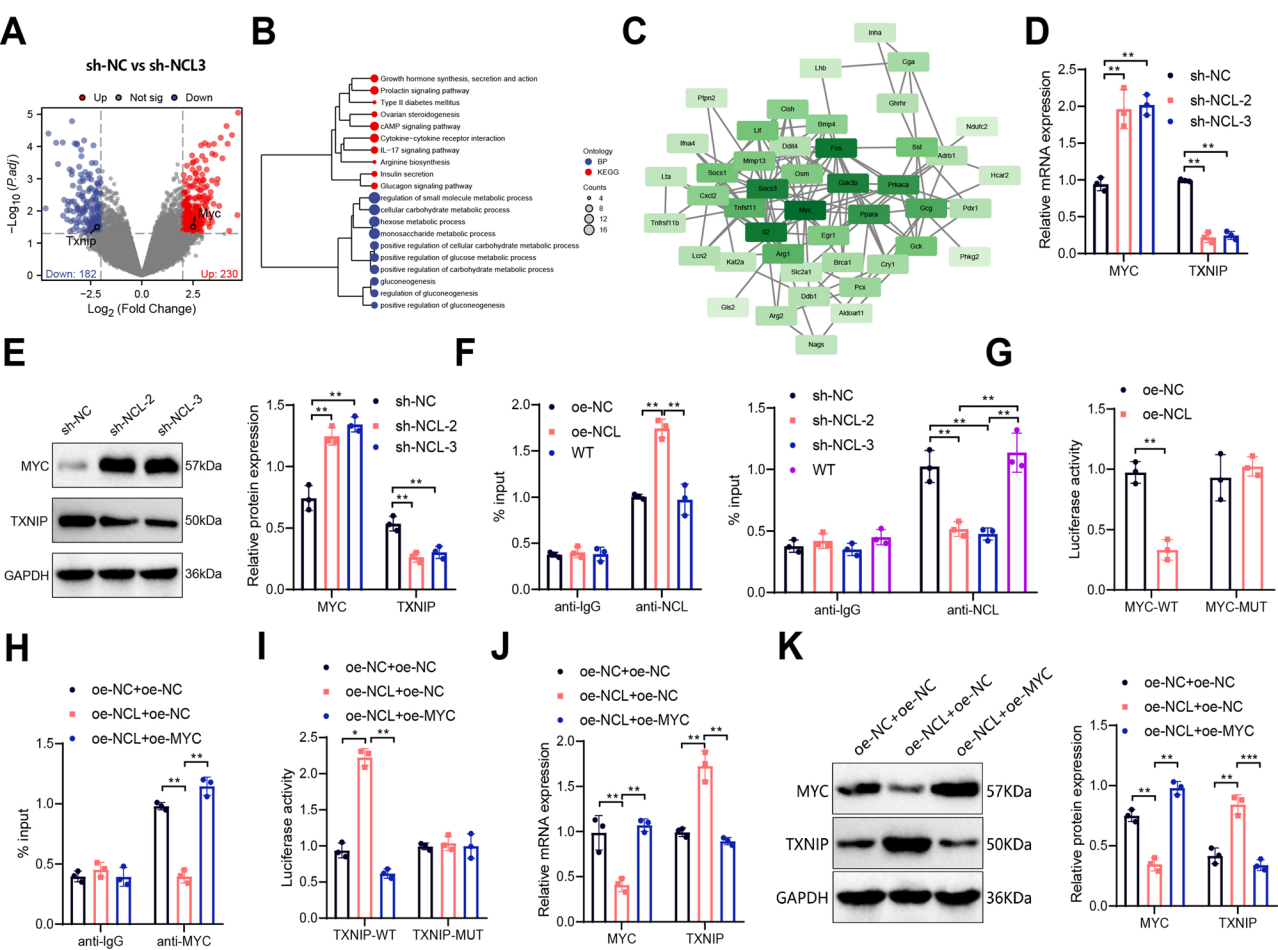
Mechanistic validation of NCL regulation of TXNIP transcription through MYC-dependent pathways. Notes: **(A)** Volcano plots depicting differential analysis of CD8^+^ T cells between the sh-NC group (*n* = 3) and sh-NCL-3 group (*n* = 3); **(B)** Clustered tree diagrams of GO and KEGG enrichment analysis of DEGs; **(C)** Protein interaction network diagram of DEGs, with core gene MYC highlighted within a black dashed box; **(D-E)** Expression levels of MYC and TXNIP in CD8^+^ T cells of the sh-NC group and sh-NCL-2 and sh-NCL-3 group measured by RT-qPCR **(D)** and Western Blot **(E)**; **(F)** ChIP experiment assessing the enrichment of NCL on the MYC promoter in each group of cells; **(G)** Dual-luciferase assay determining the MYC promoter luciferase activity in each group of cells; **(H)** ChIP experiment examining the enrichment of MYC on the TXNIP promoter in each group of cells; **(I)** Dual-luciferase assay measuring the TXNIP promoter luciferase activity in each group of cells; **(J-K)** Expression levels of MYC and TXNIP in CD8^+^ T cells of each group determined by RT-qPCR (J) and Western Blot **(K)**. All cell experiments were conducted in triplicate; * indicates *P* < 0.05, ** indicates *P* < 0.01, and *** indicates *P* < 0.001

Transcriptome sequencing of CD8^+^ T cells in the sh-NC and sh-NCL-3 groups revealed 230 upregulated genes and 182 downregulated genes, identified using |log_2_FC| > 2 and *P* < 0.05 as thresholds (Fig. 5A). GO and KEGG enrichment analyses indicated that these DEGs were predominantly enriched in pathways related to glucose metabolism (Fig. 5B). Subsequent protein interaction analysis of 57 genes involved in key pathways revealed that MYC was situated at the central node of the network (Fig. 5C). Literature evidence supports the significant role of the MYC/TXNIP axis in glucose metabolism (Li et al. 2022a, b; Lim et al. 2023; Ji et al. 2016). Transcriptome profiling showed that compared to the sh-NC group, the sh-NCL-3 group exhibited upregulation of MYC expression and downregulation of TXNIP expression (Fig. 5A). Therefore, it is postulated that NCL potentially modulates CD8^+^ T cell glucose metabolism by regulating the MYC/TXNIP axis.

Further validation through RT-qPCR and Western Blot confirmed the regulatory effect of NCL on MYC and TXNIP expression. The sh-NCL-2 and sh-NCL-3 groups showed significantly increased MYC expression and decreased TXNIP expression compared to the sh-NC group, consistent with the sequencing results (Fig. 5D-E). The results of ChIP experiments demonstrated that the overexpression of NCL led to an enrichment of NCL protein on the MYC promoter (Fig. 5F). Conversely, silencing NCL hindered the presence of NCL protein on the MYC promoter (Fig. 5F), and furthermore, endogenous NCL was found to physically bind to the MYC promoter region (Fig. 5F). Subsequent dual-luciferase reporter gene assays revealed that overexpression of NCL suppressed the activity of the MYC promoter (Fig. 5G). Similarly, ChIP and luciferase assays indicated that MYC could suppress TXNIP transcription, with MYC overexpression reversing the inhibitory effect of NCL on TXNIP transcription (Fig. 5H-I). Furthermore, simultaneous modulation of NCL and MYC expression and subsequent assessment of TXNIP expression changes showed that MYC downregulation and TXNIP upregulation were achieved in the oe-NCL + oe-NC group compared to the oe-NC + oe-NC group; conversely, in the oe-NCL + oe-TXNIP group, MYC was significantly upregulated while TXNIP was downregulated compared to the oe-NCL + oe-NC group (Fig. 5J-K), indicating a MYC-dependent promotion of TXNIP expression by NCL.

Previous studies have suggested that TXNIP serves as a negative regulator of glucose metabolism in tumor cells (Sullivan et al. 2018; Wang and Chen 2021); however, its role in CD8^+^ T cell glucose metabolism remains unclear. Therefore, overexpression of TXNIP in CD8^+^ T cells led to decreased glucose uptake, reduced lactate production, lower ECAR and OCR, decreased ATP production, decreased mitochondrial membrane potential, and downregulation of glucose metabolism enzymes GLUT1, GLUT4, HK2, LDHA, and LDHB compared to the oe-NC group (Figure S6). These findings indicate that TXNIP overexpression significantly inhibits glucose metabolism in CD8^+^ T cells.

**Fig. 6:**
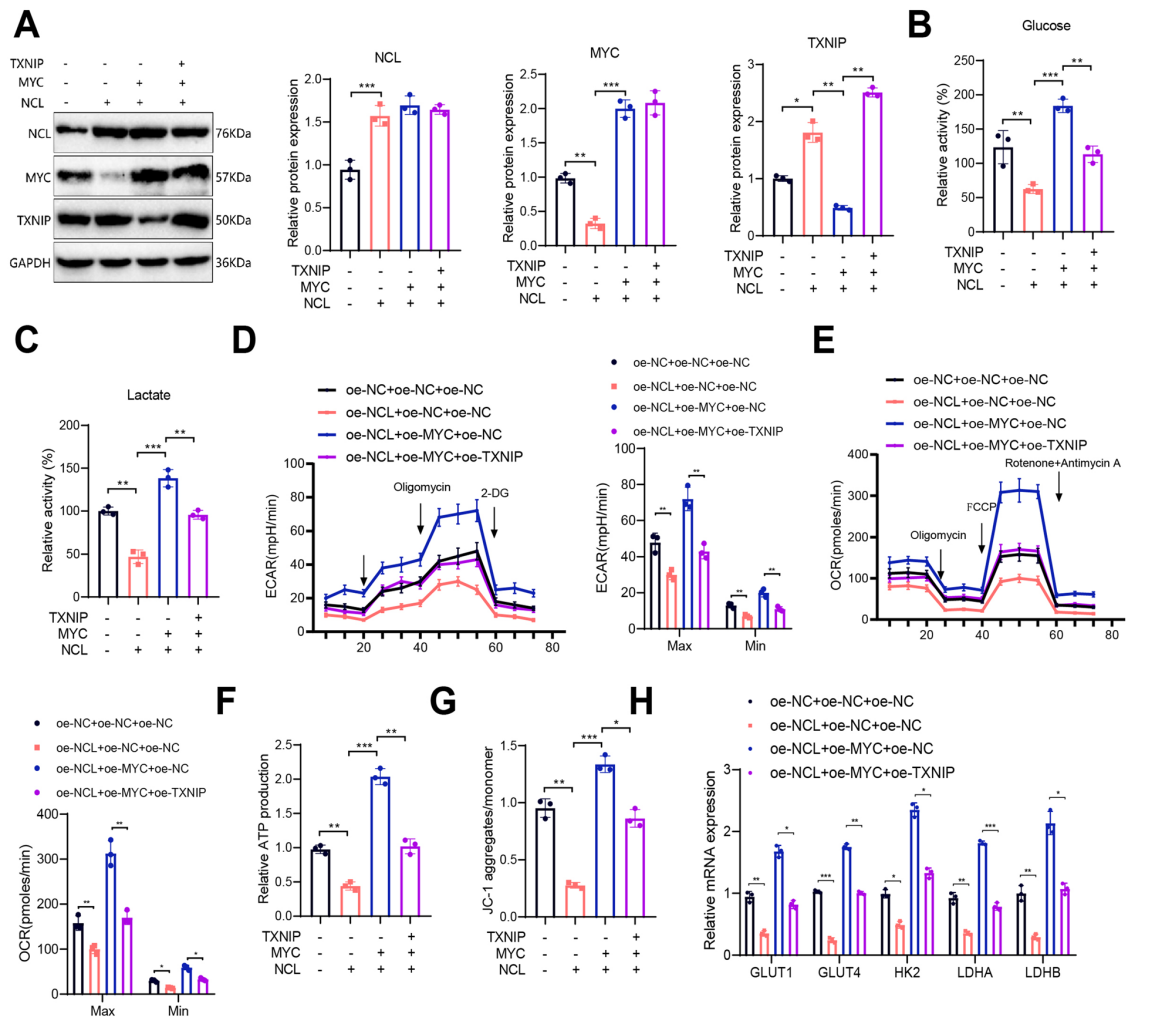
Impact of NCL targeting the MYC/TXNIP axis on the reprogramming of glucose metabolism in CD8^+^ T cells. Notes: **(A)** Protein expression of NCL, MYC, and TXNIP in CD8^+^ T cells of each group analyzed by Western Blot; **(B)** Glucose uptake levels in CD8^+^ T cells of each group; **(C)** Lactate production levels in CD8^+^ T cells of each group; **(D)** Changes in ECAR of CD8^+^ T cells in each group, including minimum and maximum values; **(E)** Changes in OCR of CD8^+^ T cells in each group, including minimum and maximum values; **(F)** Measurement of intracellular ATP in CD8^+^ T cells of each group using an ATP assay kit; **(G)** Calculation of the red-green signal ratio of JC-1 to determine mitochondrial membrane potential in CD8^+^ T cells of each group analyzed by flow cytometry; **(H)** Expression levels of GLUT1, GLUT4, HK2, LDHA, and LDHB in CD8^+^ T cells of each group assessed by RT-qPCR. All cell experiments were performed three times; * indicates *P* < 0.05, ** indicates *P* < 0.01, and *** indicates *P* < 0.001

Finally, CD8^+^ T cells were divided into four groups: oe-NC + oe-NC + oe-NC, oe-NCL + oe-NC + oe-NC, oe-NCL + oe-MYC + oe-NC, and oe-NCL + oe-MYC + oe-TXNIP, followed by measurements of glucose uptake, lactate production, ECAR, OCR, ATP production, mitochondrial membrane potential, and expression of glucose metabolism enzymes to elucidate the specific mechanism by which NCL influences CD8^+^ T cell glucose metabolism via regulation of the MYC/TXNIP axis. Results showed that compared to the oe-NC + oe-NC + oe-NC group, the oe-NCL + oe-NC + oe-NC group exhibited lower glucose uptake and lactate production, decreased ECAR and OCR, reduced ATP production, decreased mitochondrial membrane potential, and downregulated expression of GLUT1, GLUT4, HK2, LDHA, and LDHB. Additionally, compared to the oe-NCL + oe-NC + oe-NC group, the oe-NCL + oe-MYC + oe-NC group showed increased glucose uptake and lactate production, elevated ECAR and OCR, increased ATP production, higher mitochondrial membrane potential, and upregulated expression of GLUT1, GLUT4, HK2, LDHA, and LDHB. These results suggest that MYC overexpression reverses the inhibitory effect of NCL overexpression on CD8^+^ T cell glucose metabolism, while noteworthy, TXNIP overexpression can further reverse the effects induced by MYC overexpression (Fig. 6). Thus, it is proposed that overexpression of NCL releases the transcriptional suppression of TXNIP by MYC, subsequently inhibiting glucose metabolism in CD8^+^ T cells.

Overexpression of NCL Modulates CD8^+^ T Cell Immune Function by Targeting the MYC/TXNIP Axis.

**Fig. 7:**
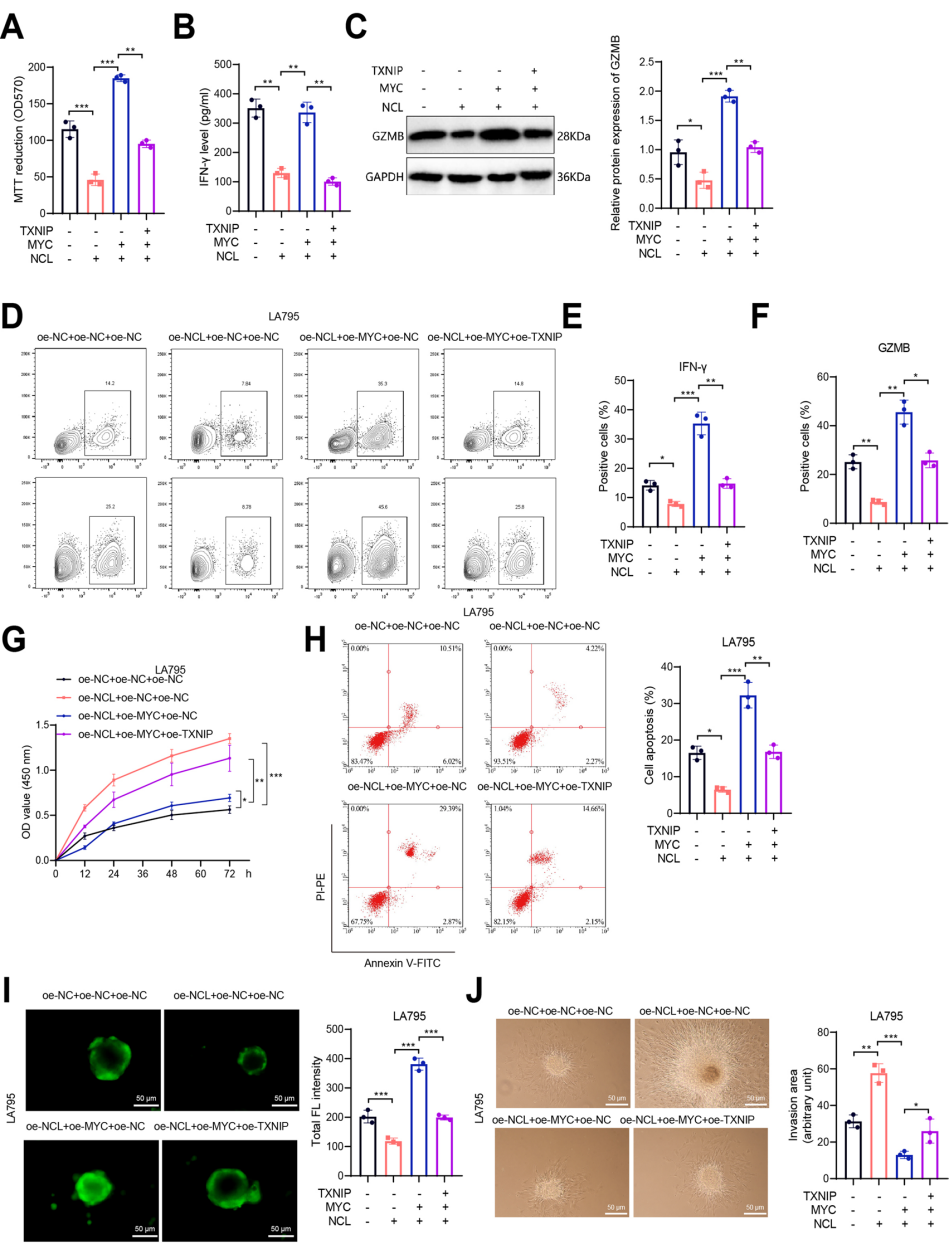
Influence of NCL targeting the MYC/TXNIP axis on CD8^+^ T cell-mediated killing of LA795 cells. Notes: **(A)** Assessment of cell viability using MTT assay; **(B)** Measurement of IFN-γ levels in cell culture supernatant via ELISA; **(C)** Quantification of GZMB levels in CD8^+^ T cells by Western Blot; **(D-F)** Analysis of the quantity of IFN-γ and GZMB in CD8^+^ T cells by flow cytometry **(D)**, with quantification of the proportion of IFN-γ **(E)** and GZMB **(F)** positive cells; **(G)** Evaluation of tumor cell proliferation at different time points using CCK-8 assay; **(H)** Analysis of tumor cell apoptosis by flow cytometry, with apoptotic cells marked with a red square box and a statistical chart of apoptotic cells on the right; **(I)** Confocal microscopy images displaying the infiltration of CFSE-labeled CD8^+^ T cells into MCS, scale bar = 50 μm, with a statistical chart of average CFSE fluorescence intensity **(FL)** in MCS on the right; **(J)** Bright-field images of cell cultures captured using inverted microscopy, with a white dashed line outlining the invasive area, scale bar = 50 μm, and a statistical chart of the invasive area on the right. All cell experiments were replicated three times; * indicates *P* < 0.05, ** indicates *P* < 0.01, and *** indicates *P* < 0.001

To investigate whether NCL alters CD8^+^ T cell immune function by targeting the MYC/TXNIP axis, we assessed the viability of CD8^+^ T cells in each group using the MTT assay. Additionally, we measured the levels of IFN-γ and GZMB through ELISA, Western Blot, and flow cytometry. Our results showed that compared to the oe-NC + oe-NC + oe-NC group, the oe-NCL + oe-NC + oe-NC group exhibited significantly reduced cell viability, markedly decreased IFN-γ secretion, a significant decrease in GZMB protein levels, and a notable decrease in the quantity of IFN-γ and GZMB-positive cells. Conversely, when compared to the oe-NCL + oe-NC + oe-NC group, the oe-NCL + oe-MYC + oe-NC group displayed enhanced cell viability, significantly increased IFN-γ secretion, elevated GZMB protein levels, and a notable increase in the quantity of IFN-γ and GZMB-positive cells. Furthermore, in comparison to the oe-NCL + oe-MYC + oe-NC group, the oe-NCL + oe-MYC + oe-TXNIP group exhibited decreased cell viability, a slight reduction in IFN-γ secretion, a slight decrease in GZMB protein levels, and a slight decrease in the quantity of IFN-γ and GZMB-positive cells (Fig. 7A-F).

These findings suggest that MYC overexpression can reverse the inhibitory effects of NCL overexpression on the activity and cytotoxicity of CD8^+^ T cells, while TXNIP overexpression can further counteract the promoting effects of MYC overexpression on the activity and cytotoxicity of CD8 + T cells.

Subsequently, CD8^+^ T cells from different groups (after activation) were co-cultured with lung adenocarcinoma cell lines LA795 or CMT64 to evaluate the proliferation capacity and apoptosis of tumor cells. 3D cancer spheroids were constructed using lung adenocarcinoma cell lines and co-cultured with CFSE-labeled CD8^+^ T cells to assess the infiltration ability of CD8^+^ T cells. Additionally, after 4 days of co-culture, the invasive spread of spheroids in each group was observed to assess the impact of CD8^+^ T cells on the invasion ability of lung adenocarcinoma.

Initially, compared to the oe-NC + oe-NC + oe-NC group, the oe-NCL + oe-NC + oe-NC group exhibited enhanced tumor cell proliferation, reduced apoptosis (Fig. 7G-H, Figure S7A-B), significantly decreased permeability (Fig. 7I, Figure S7C), and a notable increase in the invasive spread capacity of spheroids (Fig. 7J, Figure S7D). These results indicate that NCL overexpression inhibits the infiltration ability of CD8^+^ T cells, thereby promoting the growth, invasion, and survival of lung adenocarcinoma cells.

Moreover, compared to the oe-NCL + oe-NC + oe-NC group, the oe-NCL + oe-MYC + oe-NC group showed decreased tumor cell proliferation, increased apoptosis (Fig. 7G-H, Figure S7A-B), significantly increased permeability (Fig. 7I, Figure S7C), and a notable decrease in the invasive spread capacity of spheroids (Fig. 7J, Figure S7D). These observations indicate that MYC overexpression can reverse the inhibitory effects of NCL overexpression on the infiltration ability of CD8 + T cells, thus inhibiting the growth, invasion, and survival of lung adenocarcinoma cells.

Furthermore, when compared to the oe-NCL + oe-MYC + oe-NC group, the oe-NCL + oe-MYC + oe-TXNIP group exhibited enhanced tumor cell proliferation, reduced apoptosis (Fig. 7G-H, Figure S7A-B), significantly decreased permeability (Fig. 7I, Figure S7C), and a notable increase in the invasive spread capacity of spheroids (Fig. 7J, Figure S7D). These outcomes suggest that TXNIP overexpression can reverse the promoting effects of MYC on the infiltration ability of CD8^+^ T cells, thereby promoting the growth, invasion, and survival of lung adenocarcinoma cells.

Overall, these results indicate that NCL overexpression can relieve the transcriptional suppression of TXNIP by MYC, leading to decreased activity and cytotoxicity of CD8^+^ T cells, ultimately promoting the growth, invasion, and survival of lung adenocarcinoma cells.

Overexpression of NCL Inhibits Intratumoral Anti-Tumor Immunity and Glycolytic Activity in CD8^+^ T Cells by Unblocking MYC-Mediated Transcriptional Repression of TXNIP.

**Fig. 8:**
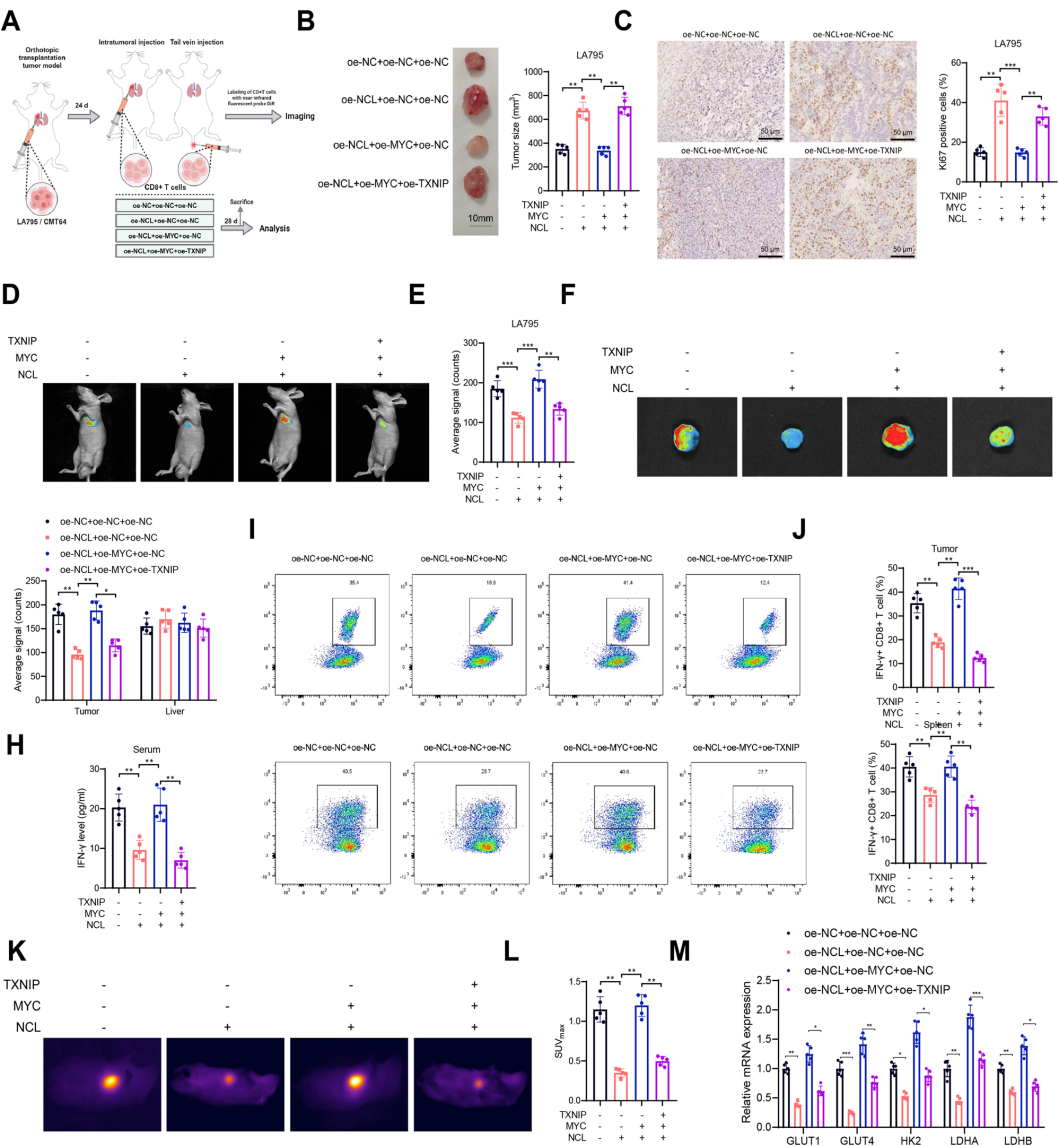
Impact of NCL targeting MYC/TXNIP axis on the in vivo anti-tumor immune response and glucose metabolism activity of CD8^+^ T cells. Notes: **(A)** Flow chart of animal experiments; **(B)** Morphological images of tumor tissues in each group of mice along with volumetric statistics; **(C)** Immunohistochemical staining images of tumor tissues in each group of mice for Ki67, scale bar = 25 μm, and statistical chart of positive cell proportion; **(D)** In vivo near-infrared imaging of mice six hours after injection of CD8^+^ T cells into tumor; **(E)** Semi-quantitative visualization of CD8^+^ T cell signals in tumors; **(F-G)** Ex vivo imaging of tumors 24 h after tail vein injection of CD8^+^ T cells **(F)**, semi-quantitative display of CD8^+^ T cell signals in tumors **(G)**; **(H)** ELISA measurement of serum IFN-γ concentration in each group of mice; **(I)** Flow cytometry analysis of the percentage of IFN-γ positive cells in CD8^+^ T cells within tumor tissues of each group of mice and columnar statistical graph; **(J)** Flow cytometry analysis of the percentage of IFN-γ positive cells in CD8^+^ T cells within splenic tissues of each group of mice and columnar statistical graph; **(K)** 18 F-FDG PET/CT images of mice in each group; **(L)** Quantitative statistics of SUVmax values in each group; **(M)** RT-qPCR analysis of the expression levels of GLUT1, GLUT4, HK2, LDHA, and LDHB in CD8^+^ T cells of each group; * represents *P* < 0.05, ** represents *P* < 0.01, *** represents *P* < 0.001, with 5 mice per group

Previous in vitro cell experiments have demonstrated that NCL regulates the anti-tumor immune function of CD8 + T cells via targeting the MYC/TXNIP axis. Thus, we further investigated the impact of this mechanism on tumor cell proliferation and invasion in vivo. Subsequently, we intrathoracically injected LA795 or CMT64 cells into nude mice to establish an orthotopic tumor model, followed by intratumoral or intravenous injection of CD8^+^ T cells into the tumor-bearing mice, divided into groups as oe-NC + oe-NC + oe-NC, oe-NCL + oe-NC + oe-NC, oe-NCL + oe-MYC + oe-NC, and oe-NCL + oe-MYC + oe-TXNIP (Fig. 8A). We observed that compared to the oe-NC + oe-NC + oe-NC group, mice in the oe-NCL + oe-NC + oe-NC group exhibited larger tumor volumes and a higher ratio of ki67-positive cells. Conversely, the oe-NCL + oe-MYC + oe-NC group demonstrated slower tumor growth, smaller tumor volumes, and a lower ki67-positive cell ratio compared to the oe-NCL + oe-NC + oe-NC group. Furthermore, the oe-NCL + oe-MYC + oe-TXNIP group showed a significant increase in tumor volume and ki67-positive cell ratio compared to the oe-NCL + oe-MYC + oe-NC group (Fig. 8B-C; Figure S8A-B). This indicates that the overexpression of NCL leads to the relief of MYC-mediated transcriptional inhibition of TXNIP, thereby promoting tumor growth in vivo.

Twenty-four hours after intratumoral injection, we used near-infrared imaging to observe the accumulation of CD8^+^ T cells within the tumors. Results showed that compared to the oe-NC + oe-NC + oe-NC group, the cell accumulation significantly decreased in the oe-NCL + oe-NC + oe-NC group, increased in the oe-NCL + oe-MYC + oe-NC group and decreased again in the oe-NCL + oe-MYC + oe-TXNIP group (Fig. 8D-E; Figure S8C-D). We further assessed the infiltration capability of CD8 + T cells in the tumor model by injecting different CD8^+^ T cell groups via the tail vein and measuring the infiltration 24 h post-injection after tumor excision. Consistent with previous findings, overexpression of NCL reduced CD8^+^ T cell accumulation, whereas MYC overexpression reversed the effects of NCL overexpression, and TXNIP overexpression further reversed the promoting effect of MYC overexpression on CD8^+^ T cell accumulation (Fig. 8F-G; Figure S8E-F).

Additionally, flow cytometry analysis of IFN-γ positive cells in CD8^+^ T cells from tumors and spleens of different mouse groups, along with ELISA measurements of IFN-γ concentrations in mouse serum, were conducted. Compared to the oe-NC + oe-NC + oe-NC group, mice in the oe-NCL + oe-NC + oe-NC group showed lower IFN-γ concentrations in serum and fewer IFN-γ positive cells in tumors and spleens. In contrast, the oe-NCL + oe-MYC + oe-NC group exhibited increased serum IFN-γ concentrations and a higher number of IFN-γ positive cells compared to the oe-NCL + oe-NC + oe-NC group. Moreover, both IFN-γ concentration and IFN-γ positive cells decreased in the oe-NCL + oe-MYC + oe-TXNIP group compared to the oe-NCL + oe-MYC + oe-NC group (Fig. 8H-J; Figure S8G-I). These results suggest that overexpression of NCL suppresses the infiltration capability of CD8^+^ T cells within the tumor microenvironment and their anti-tumor immunity by relieving MYC-mediated transcriptional repression of TXNIP.

Furthermore, we utilized 18 F-FDG PET/CT technology to reflect glucose metabolism and assessed the expression of key metabolic enzymes in CD8^+^ T cells isolated from the spleens and lymph nodes of different mouse groups using RT-qPCR. Our results indicated that compared to the oe-NC + oe-NC + oe-NC group, the SUVmax value significantly decreased in the oe-NCL + oe-NC + oe-NC group, with changes occurring predominantly at the CD8^+^ T cell injection site, accompanied by significant suppression of GLUT1, GLUT4, HK2, LDHA, and LDHB expression. This suggests that overexpression of NCL markedly inhibits the uptake of 18 F-FDG by CD8^+^ T cells, thereby suppressing their glucose metabolism activity. Conversely, the oe-NCL + oe-MYC + oe-NC group exhibited a significant increase in SUVmax value and upregulation of key enzymes like GLUT1. Lastly, compared to the oe-NCL + oe-MYC + oe-NC group, the oe-NCL + oe-MYC + oe-TXNIP group displayed a significant decrease in SUVmax value and downregulation of key enzymes like GLUT1 (Fig. 8K-M; Figure S8J-L). These findings demonstrate that overexpression of NCL inhibits CD8^+^ T cell glucose metabolism in vivo by alleviating MYC-mediated transcriptional repression of TXNIP, thereby weakening their infiltration capability in the tumor microenvironment and anti-tumor immunity, ultimately promoting tumor growth.

## Discussion

Lung adenocarcinoma, a common and deadly malignant tumor, has garnered significant attention regarding its pathogenesis and treatment strategies (Wang et al. 2023a, b; Shen et al. 2023; Sengupta et al. 2021). In this study, we focused on the relationship between the immune escape of lung adenocarcinoma cells and T cell dysfunction, with a special emphasis on the role of glucose metabolism in regulating T cell function. Additionally, we explored the potential mechanisms of the NCL protein in this context (Liu et al. 2023a, b; Shao et al. 2021). Distinguishing our research from previous studies, we placed particular importance on the involvement of NCL in regulating both glucose metabolism and immune escape processes, providing a fresh perspective for understanding lung adenocarcinoma development (Seephan et al. 2023; Chen et al. 2023).

Prior studies have established the crucial role of glucose metabolism in modulating immune cell function and the tumor microenvironment, and our research further delves into NCL’s role as a regulatory factor. Through bioinformatic analysis and in vitro and in vivo experiments, we identified that NCL likely modulates the activity of CD8^+^ T cells’ glucose metabolism by affecting the MYC/TXNIP axis, thereby influencing immune responses. This distinctive role of NCL in immune escape regulation, unlike previous studies that focused on protein factors like MYC, was demonstrated in our study (Dinh et al. 2023).

Numerous studies have indicated that NCL, as a highly phosphorylated acidic protein, exhibits increased expression in various tumors (Cheng et al. 2016; Xu et al. 2016). It directly or indirectly participates in signal transduction through diverse mechanisms, affecting tumor cell survival, proliferation, metastasis, and promoting tumor progression. Within the tumor microenvironment, CD8^+^ T cells, influenced by sustained antigenic stimulation and various signals, gradually transition into a dysfunctional exhausted state (Sun et al. 2023). This exhaustion state is accompanied by extensive transcriptional, epigenetic, and metabolic reprogramming, leading to impaired proliferation and cytotoxic capacity of CD8^+^ T cells, which may be one of the reasons for the altered expression levels of NCL in CD8^+^ T lymphocytes. Moreover, the altered levels of abundant cytokines and chemokines in the tumor microenvironment could potentially regulate the expression of NCL in CD8^+^ T lymphocytes. Nonetheless, further research and data are required to elucidate the specific mechanisms by which changes in the tumor microenvironment alter the expression levels of NCL in CD8^+^ T lymphocytes.

In vitro experiments confirmed the impact of NCL on the glucose metabolism and immune function of CD8^+^ T cells, revealing a close correlation between NCL expression levels and the activity of CD8^+^ T cells. Consistent with prior research findings, this discovery underscores the significant regulatory function of NCL in lung adenocarcinoma (Seephan et al. 2023). Notably, our in vivo experiments further confirmed the critical role of NCL in modulating immune cell function and tumor growth, providing crucial evidence for future translational medical research.

Transcriptome sequencing and experiments like ChIP not only confirmed that NCL regulates the glucose metabolism activity of CD8^+^ T cells via the MYC/TXNIP axis but also unveiled potential regulatory pathways of NCL in immune escape. This insight into potential targets for further understanding immune therapy in lung adenocarcinoma is of significant importance. By combining results from in vitro and in vivo experiments, we elucidated in detail how NCL influences CD8^+^ T cell immune responses through modulating glucose metabolism, providing essential cues for further research in this field.

Based on the data and results of this study, overexpression of NCL inhibits the glucose metabolism activity of CD8^+^ T cells, thereby weakening their anti-tumor immune effects in the tumor microenvironment, consequently accelerating the growth and development of lung adenocarcinoma. This conclusion not only aids in unraveling the mechanisms of immune escape in lung adenocarcinoma but also offers insights into potential targeted therapeutic strategies. Recent studies have suggested that NCL can enhance aerobic glycolysis by modulating hnRNPA1-mediated PKM alternative splicing (Wang et al. 2023a, b). This appears contradictory to the findings of the present study. However, it is important to note that the NCL-mediated enhancement of aerobic glycolysis discussed in the aforementioned study primarily occurs in colon cancer cells, whereas our research focuses on CD8^+^ T cells. NCL may exhibit different functional roles in various cellular environments. Additionally, within the tumor microenvironment, metabolic demands and signaling pathways in tumor cells differ from those in immune cells (Xiao et al. 2019), indicating that NCL’s actions in different environments may be influenced by extracellular signals and internal metabolic states. Complex interactions between different pathways exist, where NCL may regulate PKM2 alternative splicing and glycolytic metabolism while negatively modulating glucose metabolism in CD8^+^ T cells via the MYC/TXNIP axis. These findings warrant further in-depth experimental validation.

Beyond glycolysis and glucose metabolism, this study also elucidates NCL’s regulatory role in mitochondrial metabolism, as NCL silencing significantly increases OXPHOS in CD8^+^ T cells. Over the past few decades, it has been observed that lymphocyte activation accompanied by considerable proliferation often involves significant changes in cellular metabolism (WANG et al., 1976). Quiescent naive T cells mainly rely on oxidative phosphorylation to meet their energy demands (Chang et al. 2014). Interestingly, certain studies suggest that memory differentiation and effector cell function rely on specific metabolic pathways (Chang et al. 2013; O’Sullivan et al. 2014), highlighting the potential significance of enhanced oxidative phosphorylation in the formation of long-lived functional memory CD8^+^ T cells (van der Windt et al. 2012). Furthermore, some tumor subgroups exhibit a significant dependence on ATP oxidative phosphorylation (Weinberg and Chandel 2014). Therefore, NCL’s regulatory role in mitochondrial oxidative phosphorylation may represent a critical aspect of its involvement in the tumor immune evasion process.

Nonetheless, this study has limitations, such as the need for validation of some experimental results in a larger sample population. Future research should refine the experimental design to precisely ascertain the exact mechanism of NCL in tumor immune escape.

**Fig. 9:**
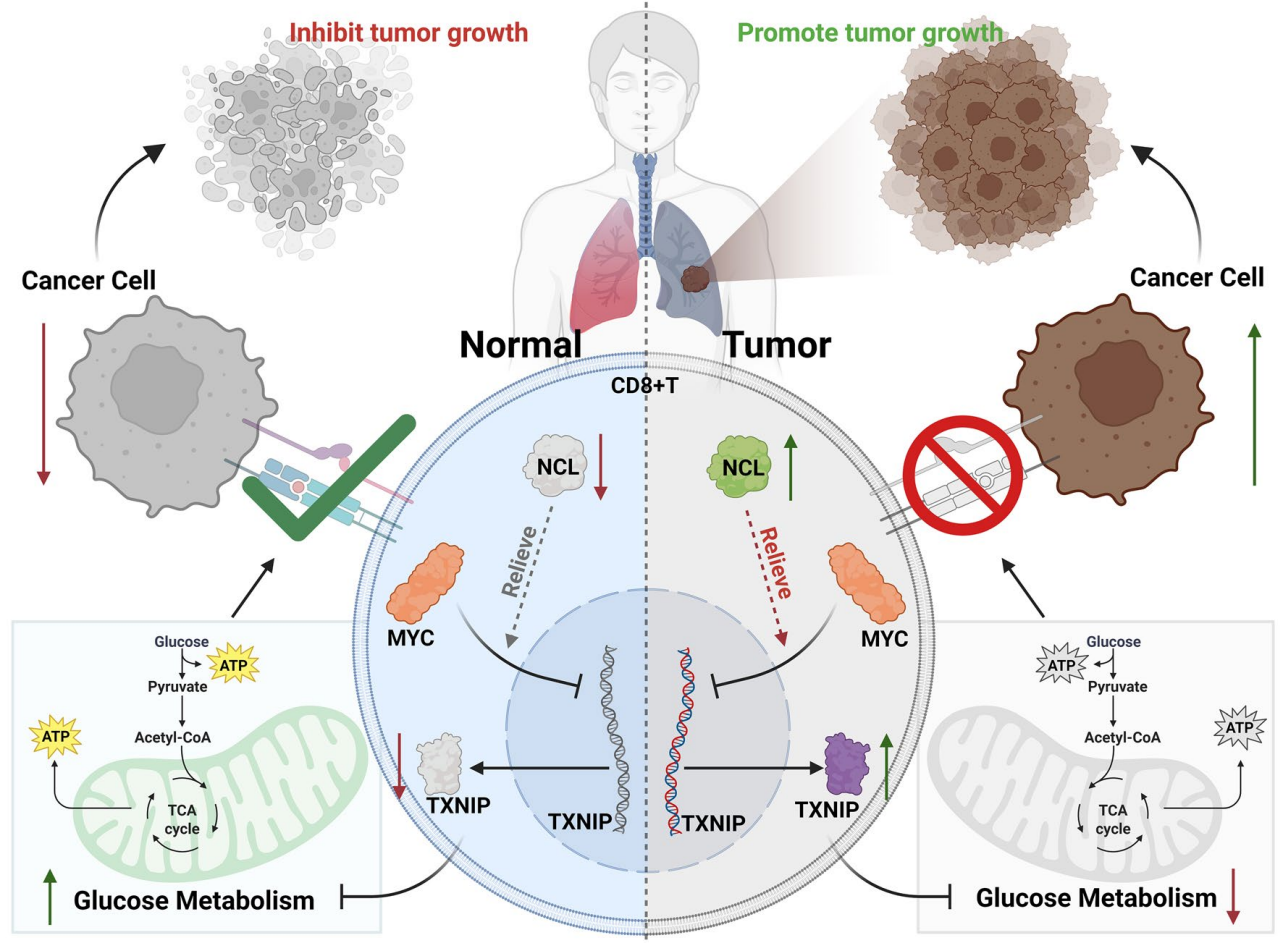
Molecular Mechanisms of NCL Targeting the MYC/TXNIP Axis to Modulate Glucose Metabolism and Anti-Tumor Immune Responses in CD8^+^ T Cells

In summary, this study delves into the role of NCL in regulating glucose metabolism and immune escape processes in lung adenocarcinoma, revealing its mechanisms and potential targets (Fig. 9). This contributes new avenues for research and possibilities for targeted therapies in lung adenocarcinoma, holding crucial scientific and clinical implications. Future research directions include further exploring the regulatory network of NCL, conducting more clinical experiments to validate research findings, and exploring more targeted treatment strategies to achieve significant breakthroughs in the treatment of lung adenocarcinoma.

## Conclusion

Based on the comprehensive evidence reviewed, it can be concluded that overexpression of NCL leads to the derepression of MYC on TXNIP transcription, inhibiting the glucose metabolism of CD8^+^ T cells. This inhibition weakens the infiltration ability of CD8^+^ T cells in the tumor microenvironment, diminishing their anti-tumor immunity and consequently promoting the occurrence and progression of lung adenocarcinoma. This study sheds light on the alteration of glucose metabolism in CD8^+^ T cells within the tumor microenvironment, offering a new perspective on the mechanisms of immune escape and therapeutic strategies for lung adenocarcinoma. Furthermore, our research suggests that NCL might serve as a novel biomarker or therapeutic target, presenting new possibilities for immune therapy in lung cancer.

While this study has yielded meaningful results, it is important to acknowledge its limitations. Primarily, the current research heavily relies on in vitro experiments and animal models, necessitating further validation of these findings’ clinical relevance through clinical samples in the future. Additionally, the role of NCL in other types of immune cells and its specific impact on immune therapy for lung adenocarcinoma require further investigation. Looking ahead, it is anticipated that more clinical studies will confirm the potential of NCL as a therapeutic target for lung adenocarcinoma, with the hope of developing treatment strategies based on key targets to enhance the survival rates of patients with this type of cancer.

## Electronic supplementary material

Below is the link to the electronic supplementary material.

Supplementary Material 1: Figure S1. Quality Control, Variance Analysis, and PCA of scRNA-seq Data. Notes: (A) Violin plots showing the distribution of the number of genes per cell (nFeature_RNA), mRNA molecule counts (nCount_RNA), and the percentage of mitochondrial genes (percent.mt) in scRNA-seq data; (B) Correlation plots between nCount and percent.mt (left) and between nCount and nFeature (right) within cells; (C) Variance analysis identifying highly variable genes in cells, with red dots indicating highly variable genes and black dots representing invariant genes; (D) Cell cycle states of each cell, where S.Score represents the S phase and G2M.Score represents the G2M phase; (E) Heatmap of the expression of constituent genes in the first two principal components; (F) Scatter plot of gene composition in the first two principal components; (G) Comparison of p-values for each principal component using the JackStrawPlot function; (H) Determination of principal components for subsequent analysis using the ElbowPlot function, identifying the inflection point based on variance changes, where important components exhibit larger standard deviations.

Supplementary Material 2: Figure S2. Scatter plot depicting the distribution of marker genes across individual cells.

Supplementary Material 3: Figure S3. Comparative Prognostic Value of GPLD1 and NCL. Notes: (A-B) The expression levels of GPLD1 (A) and NCL (B) in normal lung tissues and tumor tissues of Lung adenocarcinoma patients (Normal group, N = 59; Tumor group, N = 539); (C-D) Showing the ROC curves for predicting the survival status of Lung adenocarcinoma patients with GPLD1 (C) and NCL (D) (N = 522); (E) AUC for predicting 1-10-year survival rates of Lung adenocarcinoma patients using GPLD1 and NCL (N = 522); (F-G) Distribution of GPLD1 (F) and NCL (G) in the single-cell atlas; (H-J) Survival curves for Overall Survival (H), Disease-Specific Survival (I), and Progress-Free Interval (J) in patients with high and low GPLD1 expression levels (N = 522); *** indicates statistical significance at P <.

Supplementary Material 4: Figure S4. Validation of NCL Silencing and Overexpression Efficiency. Notes: (A) Workflow for gene silencing/overexpression and cell grouping; (B-C) Assessment of NCL expression in cells post lentivirus transfection with shRNA using RT-qPCR (B) and Western Blot (C); (D-E) Evaluation of NCL expression in cells post lentivirus transfection with NCL plasmid using RT-qPCR (D) and Western Blot (E). All cellular experiments were replicated three times, with *** indicating statistical significance at P <.

Supplementary Material 5: Figure S5. The Effect of NCL on Cytotoxicity of CD8+ T Cells against CMT64 Cells. Note: (A) Flow cytometry analysis of tumor cell apoptosis, with apoptotic cells marked by red squares; the right panel shows the statistical analysis of apoptotic cells. (B) CCK-8 assay to assess tumor cell proliferation at different time points. (C) Confocal microscopy images displaying the infiltration of CFSE-labeled CD8+ T cells in MCS; scale bar=50 μm. The panel on the right shows the statistical analysis of the average CFSE fluorescence intensity (FL) in MCS. (D) Bright-field images of cell cultures captured in the inverted microscope mode, with white dashed lines outlining the invasive cell area; scale bar=50 μm. The right panel presents the statistical analysis of the invasive area. All cellular experiments were performed in triplicate, where * indicates P <, ** indicates P <, and *** indicates P <.

Supplementary Material 6: Figure S6. Impact of TXNIP on Glucose Metabolism Reprogramming in CD8+ T Cells. Notes: (A) Glucose uptake levels in CD8+ T cells of each group; (B) Lactate production levels in CD8+ T cells of each group; (C) Changes in ECAR in CD8+ T cells of each group, along with the minimum and maximum ECAR values; (D) Changes in OCR in CD8+ T cells of each group, along with the minimum and maximum OCR values; (E) ATP levels determined by ATP assay kit in CD8+ T cells of each group; (F) Mitochondrial membrane potential assessed by flow cytometry in CD8+ T cells of each group using JC-1 signal, and calculation of the red/green signal ratio of JC-1 to determine mitochondrial potential; (G) Expression levels of GLUT1, GLUT4, HK2, LDHA, and LDHB in CD8+ T cells of each group detected by RT-qPCR. All cellular experiments were repeated three times, * indicates P <, ** indicates P <, *** indicates P<.

Supplementary Material 7: Figure S7. Impact of NCL Targeting the MYC/TXNIP Axis on CD8+ T Cell-Mediated Killing of CMT64 Cells. Notes: (A) Tumor cell proliferation at different time points assessed by CCK-8 assay; (B) Analysis of tumor cell apoptosis by flow cytometry, with apoptotic cells labeled in red squares, and the graph on the right displaying apoptotic cell statistics; (C) Confocal microscopy images showing the infiltration of CFSE-labeled CD8+ T cells in MCS, scale bar=50 μm, with the right graph depicting the average CFSE fluorescence intensity (FL) in MCS; (D) Bright-field images of cell cultures captured in inverted microscope mode, with white dashed lin

Supplementary Material 8: Figure S8. Impact of NCL Targeting the MYC/TXNIP Axis on In Vivo Anti-Tumor Immunity and Glycolytic Activity in CD8+ T Cells. Notes: (A) Morphology images of tumor tissues in mice from each group along with volumetric analysis of tumor tissue; (B) Immunohistochemical staining images (Ki67) of tumor tissues in mice from each group, scale bar = 50 µm, and proportional analysis of Ki67-positive cells; (C) In vivo near-infrared imaging of mice 6 hours after the intratumoral injection of CD8+ T cells; (D) Semi-quantitative analysis of CD8+ T cell signals in tumors; (E-F) Ex vivo imaging of tumors 24 hours post tail vein injection of CD8+ T cells (E), with semi-quantitative CD8+ T cell signal analysis in tumors (F); (G) Measurement of serum IFN-γ levels in mice using ELISA; (H) Flow cytometry analysis of the percentage of IFN-γ-positive cells in CD8+ T cells within the tumors of mice and bar chart statistics; (I) Flow cytometry analysis of the percentage of IFN-γ-positive cells in CD8+ T cells within the spleens of mice and bar chart statistics; (J) 18F-FDG PET/CT imaging of mice from each group; (K) Quantitative analysis of SUVmax in each group; (L) RT-qPCR analysis of the expression levels of GLUT1, GLUT4, HK2, LDHA, and LDHB in CD8+ T cells from each group. * denotes statistical significance at P < 0.05, ** at P < 0.01, and *** at P < 0.001, with 5 mice per group.

Supplementary Material 9

## Data Availability

The datasets used or analyzed during the current study are available from the corresponding author on reasonable request.
