# Supplementary figures and images for: The MYC/TXNIP axis mediates NCL-Suppressed CD8+T cell immune response in lung adenocarcinoma
Source: Mol Med. 2025 May 9;31:180. doi: 10.1186/s10020-025-01224-3 (PMC12063364; doi:10.1186/s10020-025-01224-3)

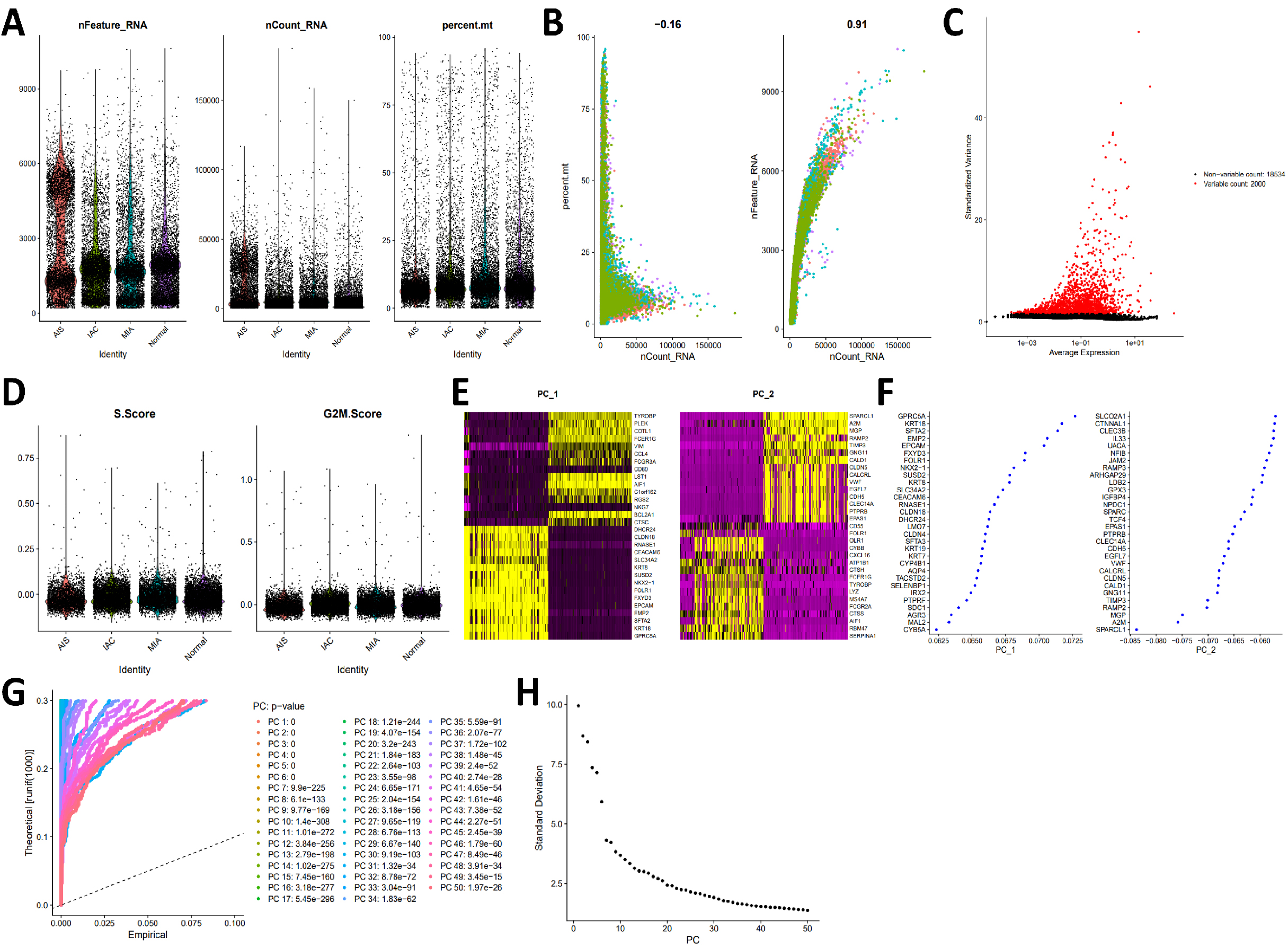

Supplement: Supplementary file 1 — Supplementary Material 1: Figure S1. Quality Control, Variance Analysis, and PCA of scRNA-seq Data. Notes: (A) Violin plots showing the distribution of the number of genes per cell (nFeature_RNA), mRNA molecule counts (nCount_RNA), and the percentage of mitochondrial genes (percent.mt) in scRNA-seq data; (B) Correlation plots between nCount and percent.mt (left) and between nCount and nFeature (right) within cells; (C) Variance analysis identifying highly variable genes in cells, with red dots indicating highly variable genes and black dots representing invariant genes; (D) Cell cycle states of each cell, where S.Score represents the S phase and G2M.Score represents the G2M phase; (E) Heatmap of the expression of constituent genes in the first two principal components; (F) Scatter plot of gene composition in the first two principal components; (G) Comparison of p-values for each principal component using the JackStrawPlot function; (H) Determination of principal components for subsequent analysis using the ElbowPlot function, identifying the inflection point based on variance changes, where important components exhibit larger standard deviations. [file 10020_2025_1224_MOESM1_ESM.jpg]

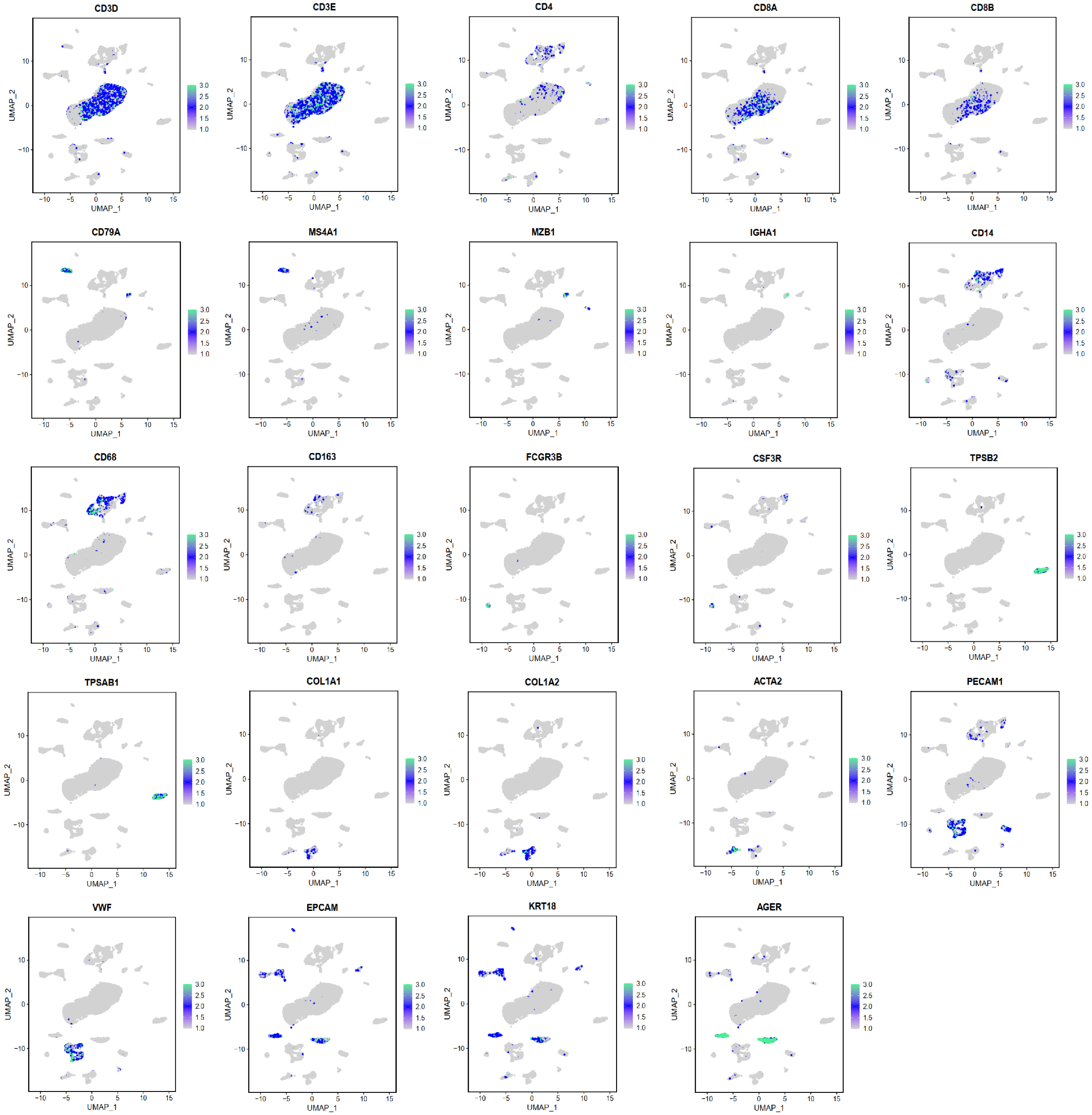

Supplement: Supplementary file 2 — Supplementary Material 2: Figure S2. Scatter plot depicting the distribution of marker genes across individual cells. [file 10020_2025_1224_MOESM2_ESM.jpg]

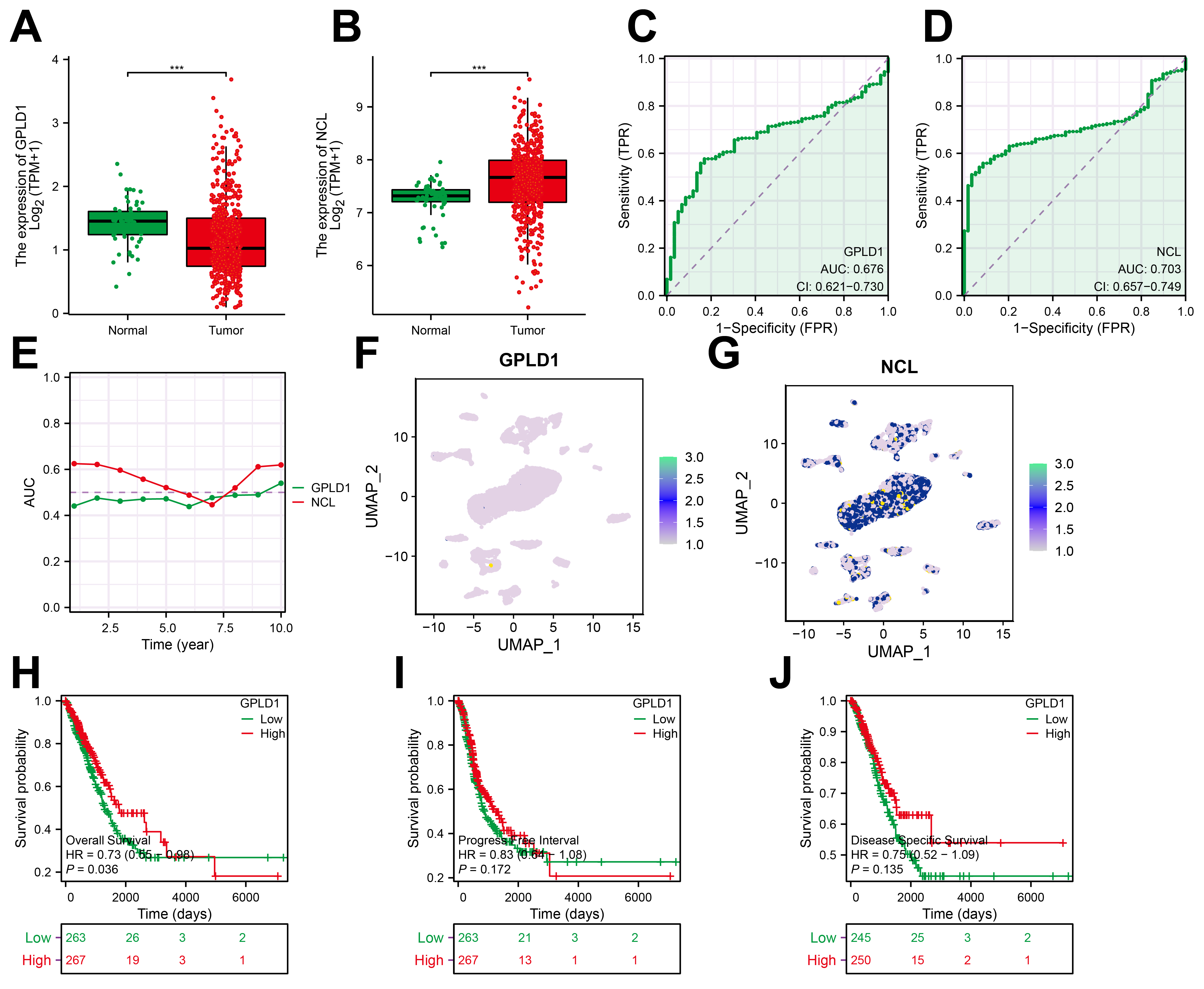

Supplement: Supplementary file 3 — Supplementary Material 3: Figure S3. Comparative Prognostic Value of GPLD1 and NCL. Notes: (A-B) The expression levels of GPLD1 (A) and NCL (B) in normal lung tissues and tumor tissues of Lung adenocarcinoma patients (Normal group, N = 59; Tumor group, N = 539); (C-D) Showing the ROC curves for predicting the survival status of Lung adenocarcinoma patients with GPLD1 (C) and NCL (D) (N = 522); (E) AUC for predicting 1-10-year survival rates of Lung adenocarcinoma patients using GPLD1 and NCL (N = 522); (F-G) Distribution of GPLD1 (F) and NCL (G) in the single-cell atlas; (H-J) Survival curves for Overall Survival (H), Disease-Specific Survival (I), and Progress-Free Interval (J) in patients with high and low GPLD1 expression levels (N = 522); *** indicates statistical significance at P <. [file 10020_2025_1224_MOESM3_ESM.jpg]

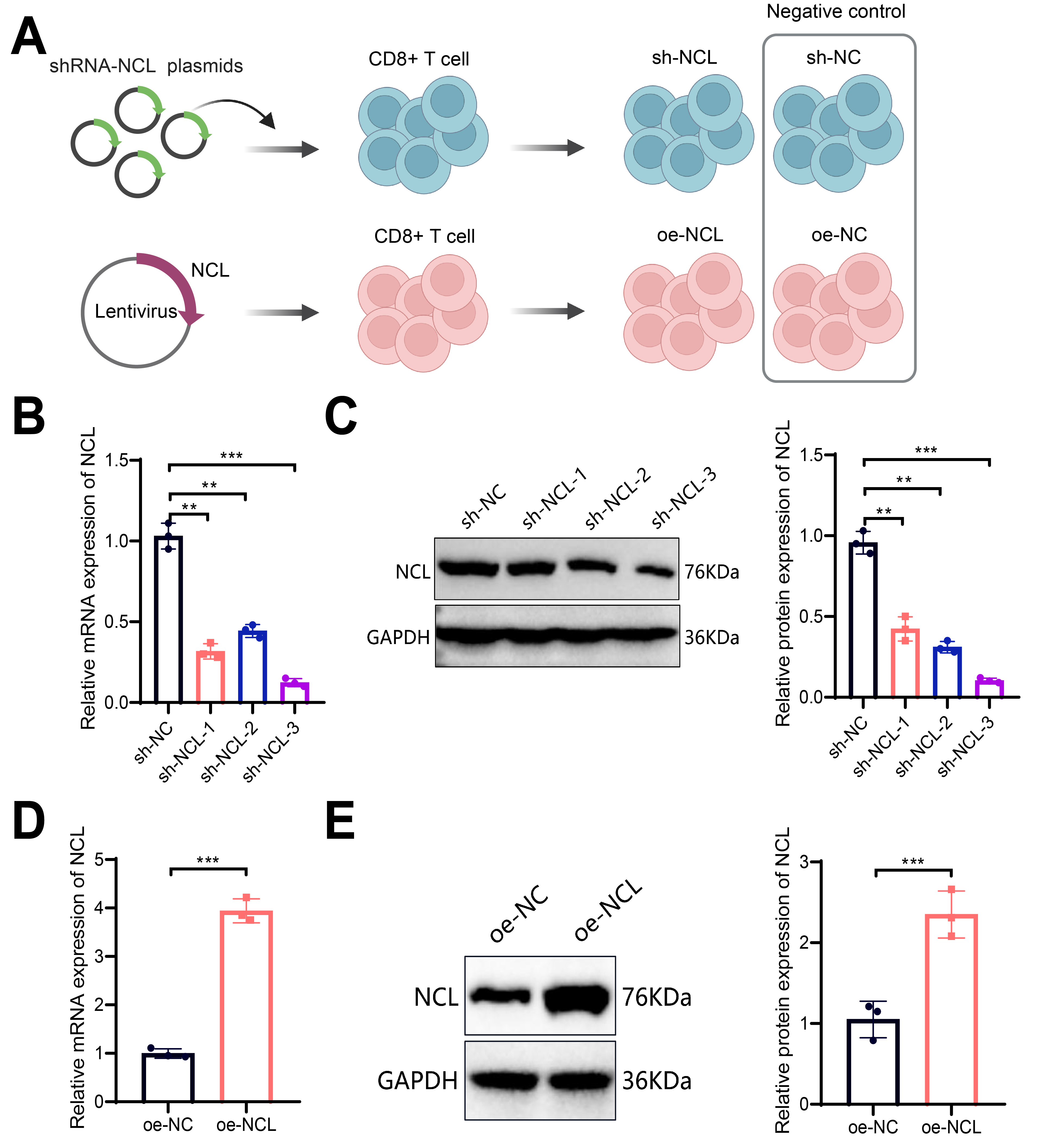

Supplement: Supplementary file 4 — Supplementary Material 4: Figure S4. Validation of NCL Silencing and Overexpression Efficiency. Notes: (A) Workflow for gene silencing/overexpression and cell grouping; (B-C) Assessment of NCL expression in cells post lentivirus transfection with shRNA using RT-qPCR (B) and Western Blot (C); (D-E) Evaluation of NCL expression in cells post lentivirus transfection with NCL plasmid using RT-qPCR (D) and Western Blot (E). All cellular experiments were replicated three times, with *** indicating statistical significance at P <. [file 10020_2025_1224_MOESM4_ESM.jpg]

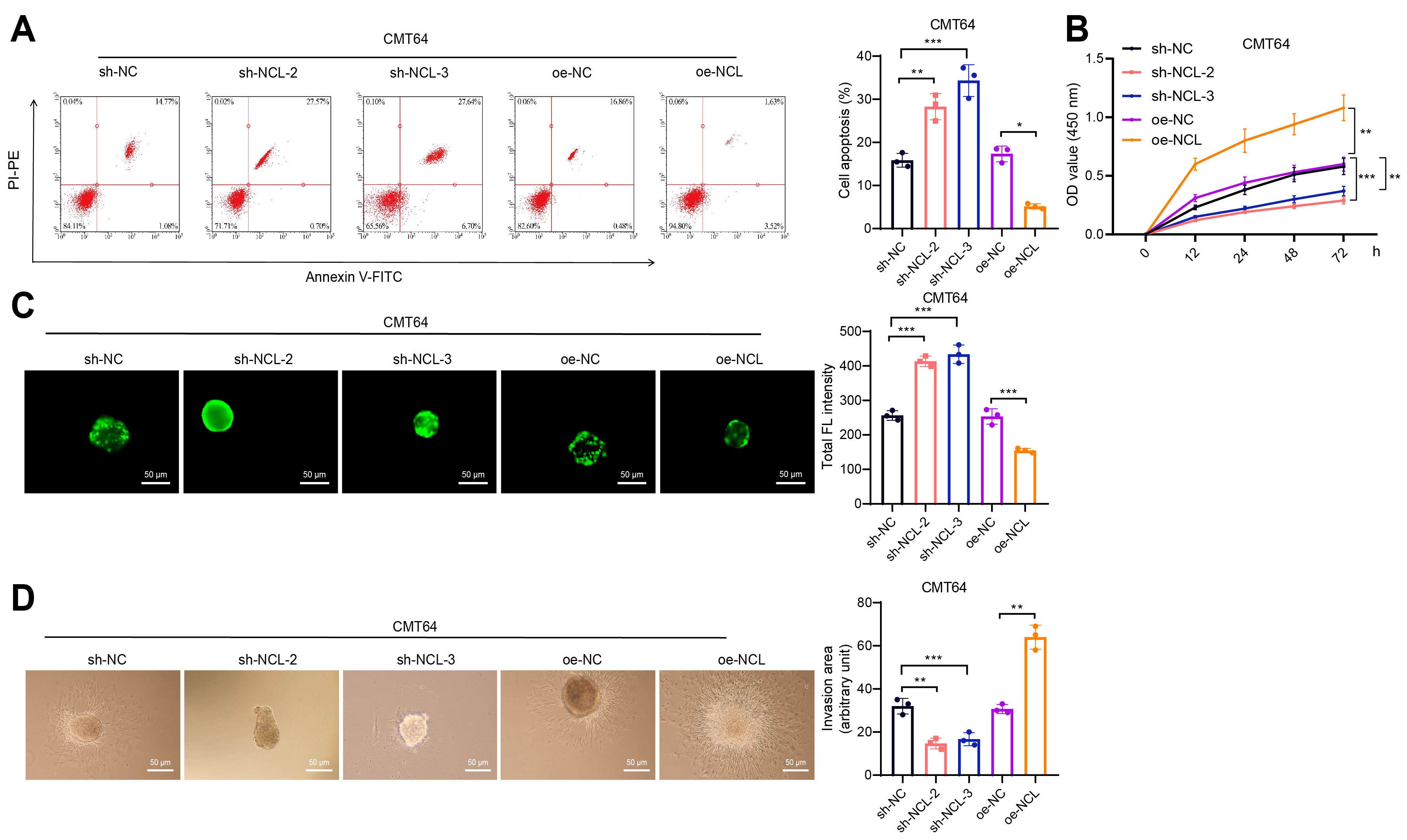

Supplement: Supplementary file 5 — Supplementary Material 5: Figure S5. The Effect of NCL on Cytotoxicity of CD8+ T Cells against CMT64 Cells. Note: (A) Flow cytometry analysis of tumor cell apoptosis, with apoptotic cells marked by red squares; the right panel shows the statistical analysis of apoptotic cells. (B) CCK-8 assay to assess tumor cell proliferation at different time points. (C) Confocal microscopy images displaying the infiltration of CFSE-labeled CD8+ T cells in MCS; scale bar=50 μm. The panel on the right shows the statistical analysis of the average CFSE fluorescence intensity (FL) in MCS. (D) Bright-field images of cell cultures captured in the inverted microscope mode, with white dashed lines outlining the invasive cell area; scale bar=50 μm. The right panel presents the statistical analysis of the invasive area. All cellular experiments were performed in triplicate, where * indicates P <, ** indicates P <, and *** indicates P <. [file 10020_2025_1224_MOESM5_ESM.jpg]

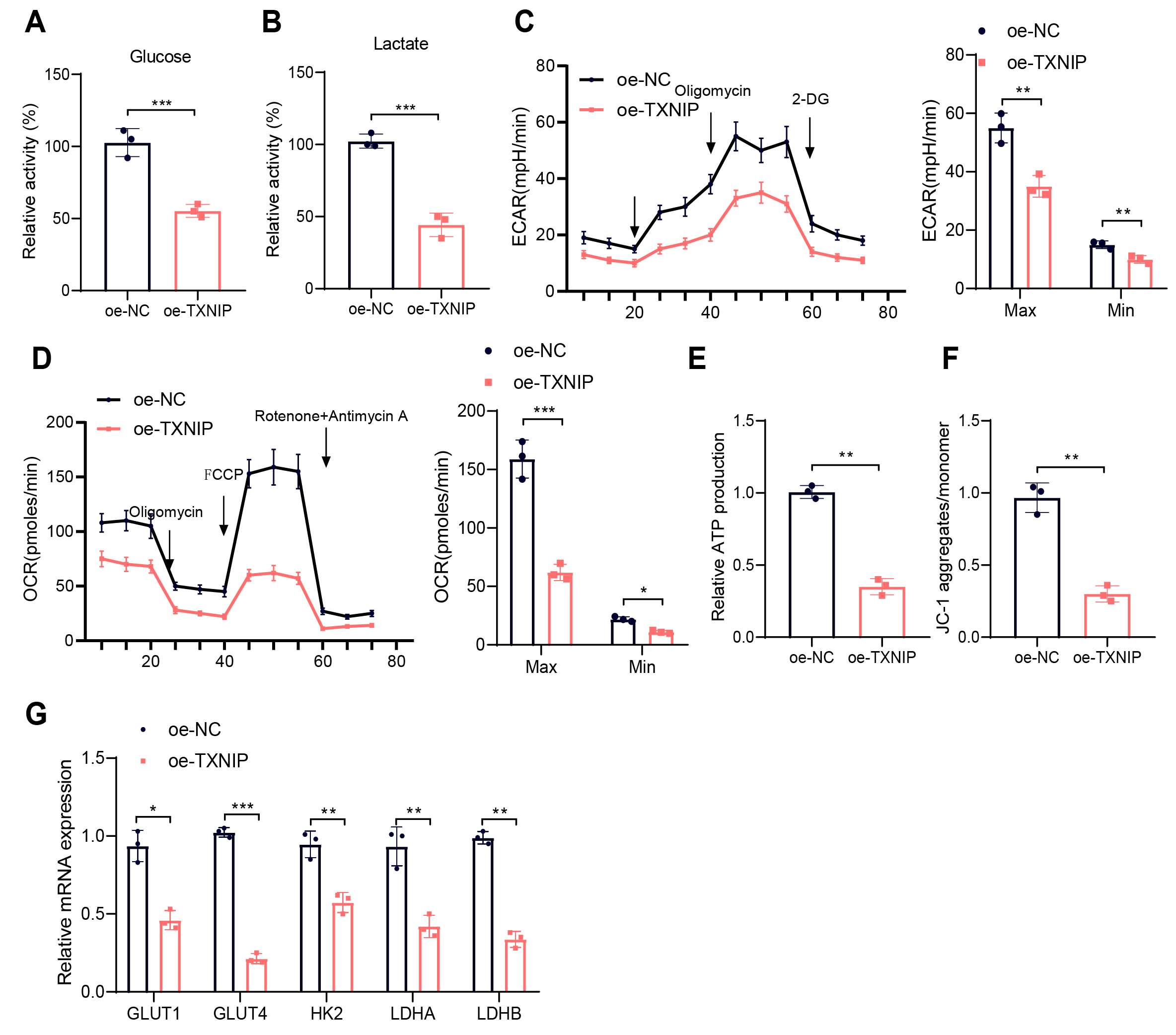

Supplement: Supplementary file 6 — Supplementary Material 6: Figure S6. Impact of TXNIP on Glucose Metabolism Reprogramming in CD8+ T Cells. Notes: (A) Glucose uptake levels in CD8+ T cells of each group; (B) Lactate production levels in CD8+ T cells of each group; (C) Changes in ECAR in CD8+ T cells of each group, along with the minimum and maximum ECAR values; (D) Changes in OCR in CD8+ T cells of each group, along with the minimum and maximum OCR values; (E) ATP levels determined by ATP assay kit in CD8+ T cells of each group; (F) Mitochondrial membrane potential assessed by flow cytometry in CD8+ T cells of each group using JC-1 signal, and calculation of the red/green signal ratio of JC-1 to determine mitochondrial potential; (G) Expression levels of GLUT1, GLUT4, HK2, LDHA, and LDHB in CD8+ T cells of each group detected by RT-qPCR. All cellular experiments were repeated three times, * indicates P <, ** indicates P <, *** indicates P<. [file 10020_2025_1224_MOESM6_ESM.jpg]

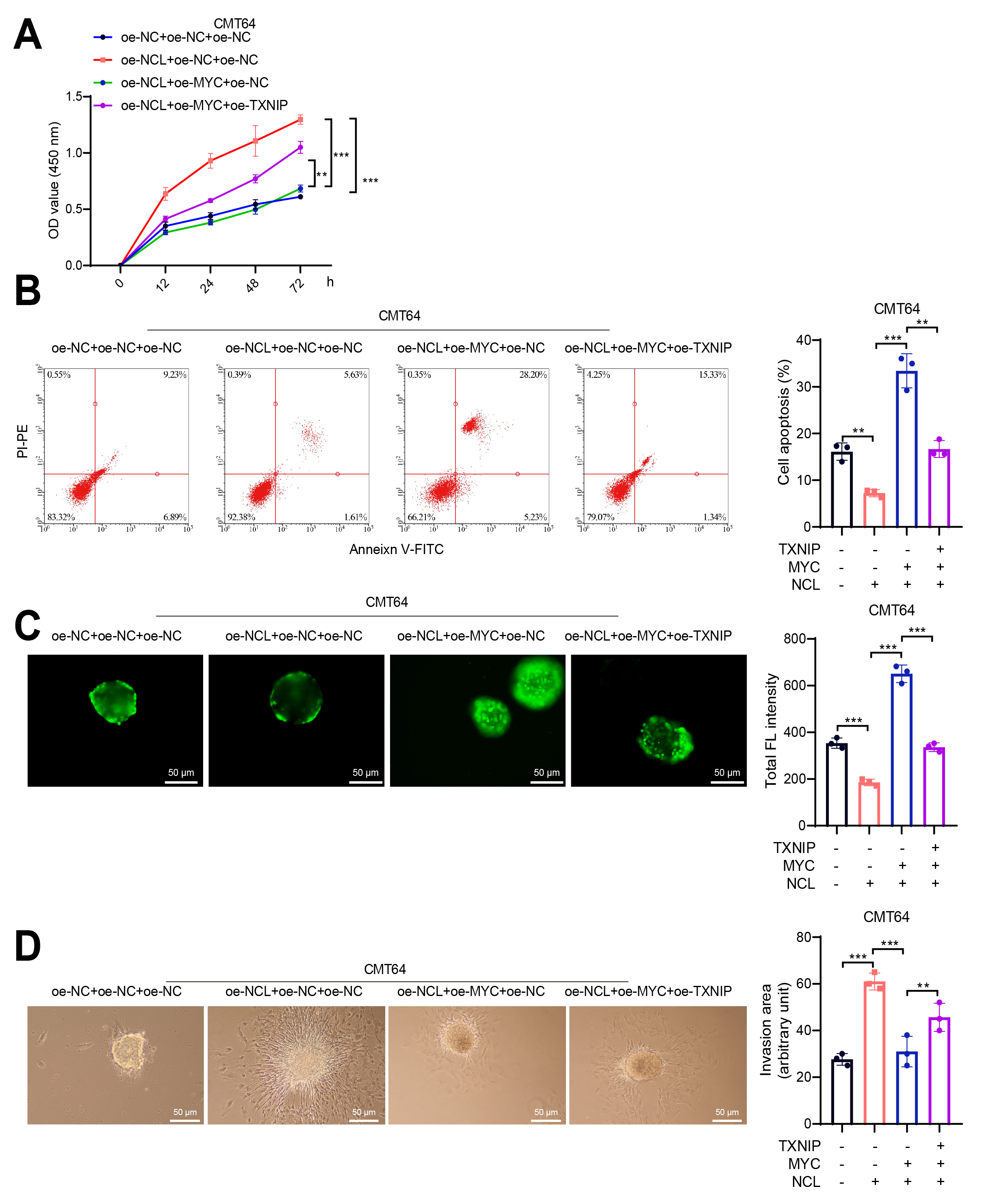

Supplement: Supplementary file 7 — Supplementary Material 7: Figure S7. Impact of NCL Targeting the MYC/TXNIP Axis on CD8+ T Cell-Mediated Killing of CMT64 Cells. Notes: (A) Tumor cell proliferation at different time points assessed by CCK-8 assay; (B) Analysis of tumor cell apoptosis by flow cytometry, with apoptotic cells labeled in red squares, and the graph on the right displaying apoptotic cell statistics; (C) Confocal microscopy images showing the infiltration of CFSE-labeled CD8+ T cells in MCS, scale bar=50 μm, with the right graph depicting the average CFSE fluorescence intensity (FL) in MCS; (D) Bright-field images of cell cultures captured in inverted microscope mode, with white dashed lin [file 10020_2025_1224_MOESM7_ESM.jpg]

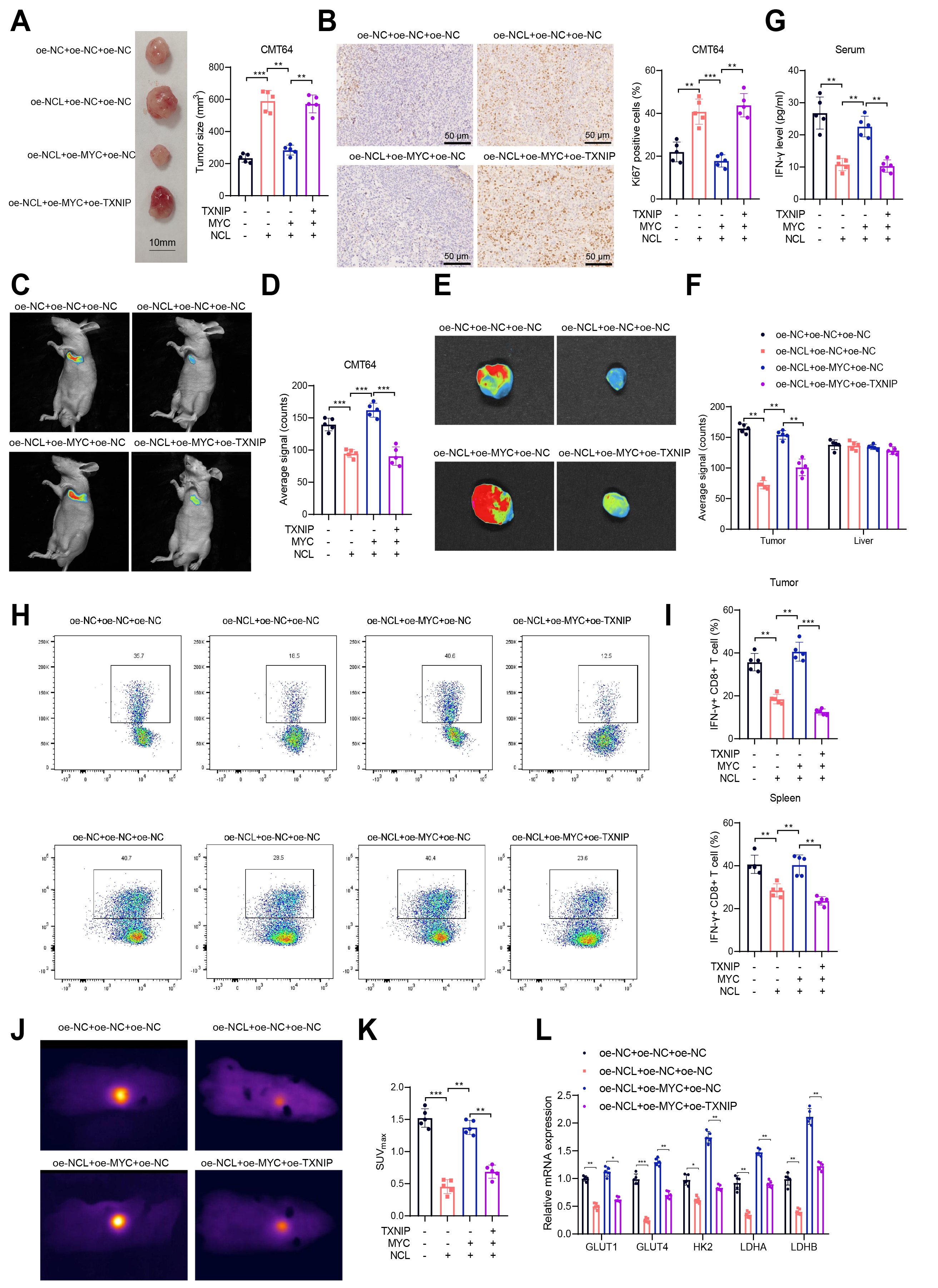

Supplement: Supplementary file 8 — Supplementary Material 8: Figure S8. Impact of NCL Targeting the MYC/TXNIP Axis on In Vivo Anti-Tumor Immunity and Glycolytic Activity in CD8+ T Cells. Notes: (A) Morphology images of tumor tissues in mice from each group along with volumetric analysis of tumor tissue; (B) Immunohistochemical staining images (Ki67) of tumor tissues in mice from each group, scale bar = 50 µm, and proportional analysis of Ki67-positive cells; (C) In vivo near-infrared imaging of mice 6 hours after the intratumoral injection of CD8+ T cells; (D) Semi-quantitative analysis of CD8+ T cell signals in tumors; (E-F) Ex vivo imaging of tumors 24 hours post tail vein injection of CD8+ T cells (E), with semi-quantitative CD8+ T cell signal analysis in tumors (F); (G) Measurement of serum IFN-γ levels in mice using ELISA; (H) Flow cytometry analysis of the percentage of IFN-γ-positive cells in CD8+ T cells within the tumors of mice and bar chart statistics; (I) Flow cytometry analysis of the percentage of IFN-γ-positive cells in CD8+ T cells within the spleens of mice and bar chart statistics; (J) 18F-FDG PET/CT imaging of mice from each group; (K) Quantitative analysis of SUVmax in each group; (L) RT-qPCR analysis of the expression levels of GLUT1, GLUT4, HK2, LDHA, and LDHB in CD8+ T cells from each group. * denotes statistical significance at P < 0.05, ** at P < 0.01, and *** at P < 0.001, with 5 mice per group. [file 10020_2025_1224_MOESM8_ESM.jpg]
