# Supplementary material for: The MYC/TXNIP axis mediates NCL-Suppressed CD8+T cell immune response in lung adenocarcinoma
Source: Mol Med. 2025 May 9;31:180. doi: 10.1186/s10020-025-01224-3 (PMC12063364; doi:10.1186/s10020-025-01224-3)
Supplement: Supplementary file 9 — Supplementary Material 9 [file 10020_2025_1224_MOESM9_ESM.docx]

**Table S1. Public Dataset Used in This Study.**

| **Dataset** | **Source** | **Type** | **Sample** |
| --- | --- | --- | --- |
| GSE164789 | GEO | scRNA-seq | One sample of normal lung tissue (Normal group) and three samples of lung adenocarcinoma tissues from patients (AIS group, MIA group, and IAC group). |
| LUAD | TCGA | RNA-seq | A total of 539 tumor tissue samples from patients with lung adenocarcinoma (including clinical information from 522 patients) and 59 adjacent non-cancerous tissue samples. |

Note: AIS refers to lung in situ adenocarcinoma, MIA refers to minimally invasive adenocarcinoma, and IAC refers to invasive adenocarcinoma.

**Table S2. shRNA Sequences.**

| **Name** | **Sequences(5'-3')** |
| --- | --- |
| sh-NC | GATGAAGAGCACCAACTC |
| sh-NCL-1 | CCTTTCCTACAGTGCAACAAA |
| sh-NCL-2 | GCAAGAACACTTCTAGCCAAA |
| sh-NCL-3 | CAAGGAAAGAAGACGAAGTTT |
| sh-independent | GCTCTGTTCGTGCAAGAATAG |

**Table S3. RT-qPCR Primer Sequences.**

| **Gene Names** | **Primer Sequences** |
| --- | --- |
| NCL (mouse) | F: 5’-TTTCCAAGTCCCACTCCCAAC-3’ |
|  | R: 5’-ATCTAATGCCCACGCCATCC-3’ |
| MYC (mouse) | F: 5’-GCTTGGCGGGAAAAAGAAGG-3’ |
|  | R: 5’-CGACCGCAACATAGGATGGA-3’ |
| TXNIP (mouse) | F: 5’-GAACCCACTCGGCTCAATCA-3’ |
|  | R: 5’-ACTGCTGAGACCCTTGCATC-3’ |
| GLUT1 (mouse) | F: 5’-ATGGATCCCAGCAGCAAGAC-3’ |
|  | R: 5’-TCATGTGACCTTAGGCCCTG-3’ |
| GLUT4 (mouse) | F: 5’-CAGATCGGCTCTGACGATGG-3’ |
|  | R: 5’-GCCCTGATGTTAGCCCTTCT-3’ |
| HK2 (mouse) | F: 5’-CTGCTTTGGAGATCCGAGGG-3’ |
|  | R: 5’-AAGCAGGCGATCATATGCGA-3’ |
| LDHA (mouse) | F: 5’-AACTTGGCGCTCTACTTGCT-3’ |
|  | R: 5’-TAGCCGCCTGAGGACTTACT-3’ |
| LDHB (mouse) | F: 5’-TCTGGACAAGATGGCAACCC-3’ |
|  | R: 5’-ATGCCGTACATTCCCTGTCC-3’ |
| GAPDH (mouse) | F: 5’-GCCTCCTCCAATTCAACCCT-3’ |
|  | R: 5’-CTCGTGGTTCACACCCATCA-3’ |

**Table S4. Information on Western blot antibodies.**

| **Target Gene Names** | **Manufacturer** | **Catalog Number** | **Dilution Ratio** |
| --- | --- | --- | --- |
| NCL (mouse) | Abcam | ab129200 | 1:10000 |
| GZMB (mouse) | Abcam | ab53097 | 1:300 |
| MYC (mouse) | Abcam | ab232691 | 1:1000 |
| TXNIP (mouse) | Abcam | ab188865 | 1:1000 |
| GAPDH (mouse) | Abcam | ab9485 | 1:2500 |
